# Heavy Metal Tolerance Genes Associated With Contaminated Sediments From an E-Waste Recycling River in Southern China

**DOI:** 10.3389/fmicb.2021.665090

**Published:** 2021-05-13

**Authors:** Shengqiao Long, Hui Tong, Xuxiang Zhang, Shuyu Jia, Manjia Chen, Chengshuai Liu

**Affiliations:** ^1^State Key Laboratory of Environmental Geochemistry, Institute of Geochemistry, Chinese Academy of Sciences, Guiyang, China; ^2^College of Earth and Planetary Sciences, University of Chinese Academy of Sciences, Beijing, China; ^3^National-Regional Joint Engineering Research Center for Soil Pollution Control and Remediation in South China, Guangdong Key Laboratory of Integrated Agro-environmental Pollution Control and Management, Guangdong Institute of Eco-environmental Science and Technology, Guangdong Academy of Sciences, Guangzhou, China; ^4^State Key Laboratory of Pollution Control and Resource Reuse, School of the Environment, Nanjing University, Nanjing, China

**Keywords:** heavy metal resistance genes, e-waste cycling, sediment, heavy metals, microbial community

## Abstract

Heavy metal pollution that results from electronic waste (e-waste) recycling activities has severe ecological environmental toxicity impacts on recycling areas. The distribution of heavy metals and the impact on the bacteria in these areas have received much attention. However, the diversity and composition of the microbial communities and the characteristics of heavy metal resistance genes (HMRGs) in the river sediments after long-term e-waste contamination still remain unclear. In this study, eight river sediment samples along a river in a recycling area were studied for the heavy metal concentration and the microbial community composition. The microbial community consisted of 13 phyla including Firmicutes (ranging from 10.45 to 36.63%), Proteobacteria (11.76 to 32.59%), Actinobacteria (14.81 to 27.45%), and unclassified bacteria. The abundance of Firmicutes increased along with the level of contaminants, while Actinobacteria decreased. A canonical correspondence analysis (CCA) showed that the concentration of mercury was significantly correlated with the microbial community and species distribution, which agreed with an analysis of the potential ecological risk index. Moreover, manually curated HMRGs were established, and the HMRG analysis results according to Illumina high-throughput sequencing showed that the abundance of HMRGs was positively related to the level of contamination, demonstrating a variety of resistance mechanisms to adapt, accommodate, and live under heavy metal-contaminated conditions. These findings increase the understanding of the changes in microbial communities in e-waste recycling areas and extend our knowledge of the HMRGs involved in the recovery of the ecological environment.

## Introduction

Electronic waste (e-waste) recycling has been a global issue for more than 10 years. Although many related countries have strict restrictions on movements of e-waste and their disposal, illegal small e-waste recycling workshops have still developed and are densely distributed in some towns of developed countries due to the economic profits and the less stringent environmental regulations. These e-waste recycling activities have inevitably resulted in severe environmental pollution (Robinson, 2009; Song and Li, 2014), such as in the towns of Longtang and Guiyu in southern China, two of the most famous e-waste recycling sites in the world (Zhang W. et al., 2012; Liu et al., 2018; Wu et al., 2019). In these areas, the typical pollutants, including polychlorinated biphenyls (PCBs), polychlorinated dibenzo-p-dioxins, polybrominated diphenyl ethers (PBDEs), and especially heavy metals, are released into local aquatic and terrestrial ecosystems, inducing the overexpression of resistance genes through the change of microbial communities in soils or sediments (Luo et al., 2011; Chen S.J. et al., 2014). The river sediments in the e-waste recycling area appear to be the major sinks for these pollutants due to the runoff of the surface water after integrating the wastes (Wang et al., 2009), especially in southern China with annual rainfall of approximately 1,700 mm. The release of these pollutants from the sediments imposes a major threat to food security, ecosystems, and human health.

Heavy metals are ubiquitous and persistent in river sediments of e-waste recycling areas and greatly affect microbial communities (Rasmussen and Sørensen, 1998; Brandt et al., 2010; Zhang et al., 2019). These indigenous microbes are crucial to the biogeochemical cycling of nutrient elements and the functioning of aquatic and terrestrial ecosystems. Due to the sensitivity to heavy metals, the diversity and abundance of microorganisms change significantly in response to different types and concentrations of heavy metals (Liu et al., 2018; Li et al., 2020). Previous studies have shown that heavy metals can damage microbial metabolism and reduce enzyme activity, leading to a decrease the diversity of the microbial community (Wang et al., 2007; Sullivan et al., 2013; Chen J. et al., 2014). The sediment microbial community plays an important role in stabilizing ecosystem functions (Jones and Lennon, 2010). Thus, an alternation in microbial community diversity and structure due to heavy metals can indirectly affect aquatic ecological functions, which are a sensitive and comprehensive indicator of aquatic and sediment environmental quality (Jones and Lennon, 2010; Zhang K. et al., 2012; Adekunle et al., 2019). A thorough knowledge of the effects of heavy metal pollution on microbial community diversity and structure will help to obtain insight into the natural attenuation process of pollutants and ecological environment recovery.

In addition, the microbial community possesses a variety of resistance mechanisms to counteract heavy metal stress, possibly due to their different resistance genes and resistance systems (Nies, 2003; Li et al., 2017; Hemmat-Jou et al., 2020). This mechanism might be correlated with the antibiotic resistance that has a significant impact on microbial ecology and environmental health (Peltier et al., 2010; Zhao and Dang, 2011). Therefore, it is important to disclose the microbial resistance mechanisms to gain more insight into the microbial response during the long-term heavy metal stress sediments in e-waste recycling areas. Recently, Xie et al. (2011) developed a microarray to analyze the microbial functional diversity of acid mine drainage from copper mines that included an abundance of some heavy metal resistance genes (HMRGs). (Li et al. (2011b) used quantitative real time PCR to investigate the relationship between the nickel concentration and the resistance gene abundance in a sequencing batch reactor. However, the previous investigations were conducted based on a limited selected subset of HMRGs that used specific primers or probes, making it impossible for a comprehensive characterization of the microbial community structure.

Previous studies have confirmed that high-throughput sequencing is a useful tool to analyze comprehensive microbial function and structure in various environments (Pachter, 2007; Zheng et al., 2019; Hemmat-Jou et al., 2020). By annotating millions of sequencing reads against a corresponding database, various antibiotic resistance genes have been identified from the environmental metagenome of activated sludge, contaminated rivers, and mine soils (Li et al., 2011b; Zhang et al., 2011; Hemmat-Jou et al., 2020). There does not exist a specialized HMRG database, which makes it nearly impossible to explore the occurrence of HMRGs in environmental metagenomes using high-throughput sequencing. Therefore, it is necessary to develop an advanced method to comprehensively overview the HMRGs residing in environmental microorganisms in river sediments of e-waste recycling areas. This is necessary to compare the microbial diversity of different samples along environmental gradients, and to evaluate the unique dominant bacterial populations in these special environments. In this study, a manually curated HMRG database is established that can identify HMRGs by retrieving the annotated sequences and related information from a public comprehensive database. Then, sediment samples are collected at different locations from a heavy metal-contaminated river in an e-waste recycling area, and the microbial communities of the sediments are described using 454 pyrosequencing. Subsequently, Illumina high-throughput sequencing is applied to determine the diversity and abundance of the HMRGs in the sediment metagenomes. This study is an effort to focus on HMRGs using a metagenomic approach, which might be technologically helpful for comprehensive characterization of microbial heavy metal resistance in an environment and the relationship between HMRGs and heavy metal contaminants.

## Materials and Methods

### Sample Collection, DNA Extraction, and Determination of Heavy Metal Concentrations

**FIGURE 1:**
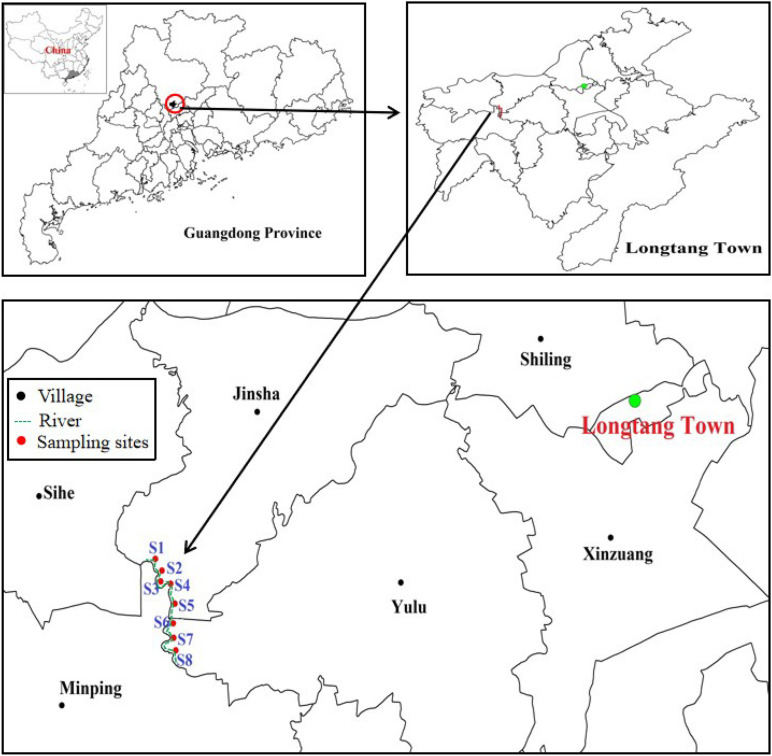
Map of sampling locations along the river at Longtang town, Guangdong Province, China. The longitude and latitude for all samples were shown in Supplementary Table 1.

Longtang town (23°32′–23°36′ N and 113°1′–113°3′ E) is located in Qingyuan city, Guangdong Province, southern China, which was once used for e-waste processing operations. Because of uncontrolled e-waste processes such as open burning and acid washing, the environment in this area, including air, water, soil, and sediment, has been seriously polluted by heavy metals (Li et al., 2011a; Wang et al., 2014). In 2012, eight sediment samples were collected from a polluted river in Longtang town along which most of the recycling operations take place (Figure 1 and Supplementary Table 1). The sediment samples were grasped at the depth of 10–15 cm below the surface of the river and kept in a mobile refrigerator at 4°C before being transferred to a laboratory.

Approximately 200 mg sediments were obtained by centrifugation to extract genomic DNA using the FastDNA Soil Kit (MP Biomedicals, OH, United States). The DNA concentration was determined by micro spectrophotometry (NanoDrop^®^ ND-1000, DE, United States) and agarose gel electrophoresis (1%). The concentrations of the heavy metals, such as arsenic (As), cadmium (Cd), chromium (Cr), copper (Cu), lead (Pb), mercury (Hg), nickel (Ni), and Zinc (Zn), were measured by inductively coupled plasma-atomic emission spectrometry (Perkin Elmer Optima 3300DV, CA, United States) after acid digestion of approximately 200 mg of ground air dried sediment samples for 32 h using concentrated nitric acid and perchloric acid (1/4, v/v) (Jiang et al., 2017).

### Assessment of the Heavy Metal Pollution

The potential ecological risk index (RI) was used to evaluate the pollution of heavy metals in the soils and sediments (Hakanson, 1980; Ma et al., 2020). RI is based on the concentration, toxicity, and background value of heavy metals, and is calculated as follows:

RI=ΣEahndE=hTCh/hB,h

where *T*_*h*_, *C*_*h*_, and *B*_*h*_ represent the standardized response coefficient for the toxicity, the measured concentrations, and the background values of heavy metals, *h*, respectively; *E*_*h*_ is the potential ecological index of the signal heavy metal, *h*; and the corresponding coefficients of As, Cd, Cr, Cu, Pb, Hg, Ni, and Zn are 10, 30, 2, 5, 5, 40, 5, and 1, respectively (Hakanson, 1980). The grading standards of the RI and *E*_*h*_ of the heavy metals are shown in Supplementary Table 2.

### Database Construction

The diversity of sequences, types, and resistance mechanisms of the HMRGs made using the HMRG construction is a large effort- and very time-consuming. To analyze and validate the data, a database of the As, Cd, and Cu resistance genes was separately created by manually retrieving all the related sequences from the National Center for Biotechnology Information (NCBI) non-redundant protein databases using key words, such as “As resistance protein” or “arsenic tolerance protein.” In detail, if the feature “note” of the coding region was defined as a confirmed heavy metal resistance protein, the sequence would be added into the database as a member. Finally, vector sequences, synthetic constructs, and redundant genes were manually removed, and the filtered database resulted in 12,221 non-redundant amino acid sequences encoding resistance to As (324 sequences), Cd (2,089 sequences) and Cu (9,808 sequences).

### Illumina High-Throughput Sequencing and 454 Pyrosequencing

The obtained genomic DNA was then submitted to the Illumina high-throughput sequencing (samples S1 and S7) and 454 pyrosequencing (samples S1–S8), which were performed using the Illumina Hiseq 2000 and FLX Titanium platform of Roche 454 from the Beijing Genome Institute (Shenzhen, China), respectively. The bacterial 16S rRNA gene (V3-V4 region, approximately 460 bp) was carried out with Illumina-specific fusion primers V3F and V4R (Claesson et al., 2009). PCR amplification was conducted with previous report (Huang et al., 2014). The 12-bp barcode with primers was used to assign individual sequences to samples. The 16S rRNA gene amplicons were submitted to an Agilent 2100 Bioanalyzer (Agilnet, United States) before using FLX Titanium platform of 454 pyrosequencing (Roche, United States). The GenBank accession numbers for the genomic datasets in NCBI are SRX825942 and SRX825518.

### Detection of the HMRGs in the Sediments

In order to determine the heavy metal resistance in the sediment in detail, all high-quality sequences were compared against the established protein databases using BLASTx^1^ with an *E*-value cut-off at 10^–5^ (Zhang et al., 2011). A read was annotated as a heavy metal resistance gene according to its best BLAST hit if (1) the similarity was greater than 95% and (2) the alignment length was at least 25 amino acids.

### Microbial Community Analysis

All the raw sequences generated by 454 pyrosequencing were first submitted to the Ribosomal Database Project (RDP) to determine the taxonomic classification of eight different samples using the specific barcodes and remove sequences shorter than 150 bps or that contained ambiguous “N” (Kristiansson et al., 2011). The obtained sequences were then subjected to denoising using the “pre.cluster” command and filtering out of the PCR chimeras using “chimera slayer” in the Mothur platform^2^ (Claesson et al., 2009; Huse et al., 2010; Roeselers et al., 2011). Reads that were regarded as the PCR chimeras were resubmitted to the RDP to confirm the accuracy, and sequences assigned to any known genus with 90% confidence were merged with the non-chimera reads to form the collection of effective sequences for each sample (Zhang T. et al., 2012). The non-bacterial sequences were automatically removed by using RDP Classifier and a self-written script. Finally, a single composite sample was normalized in equimolar amounts to produce same sequencing depth with 7,395 sequences from all samples. Phylogenetic classification of each sample was conducted by using RDP’s Classifier with a bootstrap cutoff of 50%, and the sequences were assigned to different taxonomic levels including phylum, class, order, family and genus (Zhang T. et al., 2012). Based on the operational taxonomic units (OTUs) generated by the RDP Classifier, the diversity indices of each sample including the Chao, ACE, Shannon and Simpson indexes were calculated using Mothur at a 3% distance. A heatmap based on the abundance of genera was also performed to explore the similar samples using R software and the Vegan package. A canonical correspondence analysis (CCA) was computed to determine the correlations between the heavy metal concentration and the microbial community structures.

## Results and Discussion

### Contaminations of Heavy Metals in the River Sediments

In the study, eight typical heavy metals, including As, Cd, Cu, Cr, Hg, Ni, Pb, and Zn, were determined to study the heavy metal contamination. The concentrations of the various heavy metals in the different sediment samples are presented in Supplementary Table 3. Generally, the contents of all the heavy metals in the eight samples greatly exceeded the values of the Environmental Quality Standard (EQS) for soils regulated by the Environmental Protection Agency of China and the background values in China (Supplementary Table 3). Cu had the highest concentration in all sediment samples, ranging from 4431.04 to 101128.50 μg/g, followed by Zn and Pb. Compared with the EQS values, Cu concentrations exceeded at least 224 and 5,013 times at sampling sites S8 and S1, respectively. The highest concentration of Cu was consistent with the primary business of Cu recovery in this e-waste recycling area (Zhang K. et al., 2012). Contamination with Cd, Hg, Pb, and Zn was also serious, with concentrations at least ten times higher than the maximum permissible concentration according to EQS. Generally, the concentrations of the heavy metals showed a decreasing tendency away from the source of pollution and along the river flow (Zhao et al., 2020), in which S1 had the highest concentration of each metal except for Hg and Pb. The average concentrations of all eight measured metals were higher than those found in other heavy metal-contaminated sediments (Chen and Kandasamy, 2008; Gao and Chen, 2012; Liu et al., 2018; Li et al., 2020). Due to the persistence and bioaccumulation characteristics of heavy metals in sediments (Demirak et al., 2006; Uysal et al., 2009), a high-concentration of heavy metals in the river sediments poses a serious ecological risk, and is a particular health threat to aquatic animals and humans through the food chain (Liu et al., 2014; Monroy et al., 2014; Kumar et al., 2019).

Additionally, the influence on the sediment ecosystem exerted by heavy metals and persistence time of contamination also is embodied at the molecular and community levels (Bodour et al., 2003; Grandlic et al., 2006). For example, the release of heavy metals from contaminated sediments has been shown to have an effect on microbial community diversity, activity and biomass in sediments and soils (Zhu et al., 2013; Chen J. et al., 2014). To a certain extent, a change in microorganisms in sediments reflects the entire health and condition of an ecosystem. The RI is based on the sensitivity of organisms to heavy metals (Zhu et al., 2013; Chen J. et al., 2014; Wang et al., 2018). The RI index showed that the sediments were seriously contaminated by heavy metals (Table 1). The RI index value at S1 near the source of the pollution was significantly higher than those at other sites. *E*_*i*_ for a signal heavy metal of Cd, Cu, and Hg were highest in all samples, ranging from 1086.0 to 43427.0, corresponding to the “serious” risk grades (Table 1). *E*_*i*_ of As, Cr, and Ni in all the samples decreased with the distance from the downstream along the river, reaching a “low” risk grade largely due to the low concentrations and response coefficients of the toxicity.

**TABLE 1:** Potential ecological indices (*E*_*i*_) for heavy metals and potential ecological risk index (RI) of heavy metals in all samples.

**Name**	**Potential ecological risk indices for single heavy metal (*E*_*i*_)**									
	**As**	**Cd**	**Cu**	**Hg**	**Ni**	**Cr**	**Pb**	**Zn**	**RI**
S1	274.4	43427.0	24786.4	5849.1	59.1	27.7	293.9	86.4	74804.0
S2	80.5	27356.8	2185.0	11313.2	48.8	16.4	224.0	39.1	41263.7
S3	111.3	16597.3	1347.0	2996.2	36.0	4.8	175.0	42.9	21310.4
S4	93.4	38845.9	1922.7	4196.2	49.5	18.1	309.8	73.5	45509.2
S5	54.0	15089.2	1395.1	4075.5	29.8	10.8	153.8	46.3	20854.5
S6	29.8	9048.6	1086.0	4279.2	29.9	9.9	158.0	41.3	14682.8
S7	41.0	23918.9	1695.4	7849.1	42.9	15.0	193.8	83.1	33839.3
S8	31.3	17764.9	1111.5	2528.3	33.0	3.8	86.3	47.2	21606.2

### Microbial Diversity and Taxonomic Composition in the River Sediments

The microbial community structures of the eight sediment samples were investigated using pyrosequencing of the 16S rRNA gene fragments. The microbial diversity analysis showed that the number of OTUs ranged from 1,908 (S4) to 2,528 (S3) among the samples. Samples S1, S2 and S4 had fewer OTUs than the other samples, indicating low bacterial richness in these samples. This agreed with the results of the Chao1, ACE estimation, Shannon, and Simpson indices (Supplementary Table 4). With the structural and functional resilience of microbial communities and adaptation to heavy metal contamination (Brandt et al., 2010), the diversity and richness of the bacteria showed a generally stable trend in the last four sampling sites. Hence, the diversity and richness of the bacteria were not linearly related to heavy metals in this research. Therefore, these results suggested that heavy metal contamination had a limited impact on microbial communities, which was consistent with previous study (Zhu et al., 2013). Similarly, it has been indicated that significant variations in metal concentrations changed the microbial community very little using denaturant gradient gel electrophoresis (DGGE) (Bouskill et al., 2010). However, the microbial diversity based on the relative intensity of the DGGE band and high-throughput sequencing analysis showed a decreasing trend under long-term heavy metal pollution (Jiang et al., 2017; Salam and Varma, 2019). In general, the relationship between the community diversity and heavy metal concentrations was ambiguous due to the various environmental factors. For example, previous studies have shown that nutrient concentrations and soil properties seemed to play a principal role in promoting diversity in highly metal contaminated sediments (Bouskill et al., 2010; Jiang et al., 2017).

**FIGURE 2:**
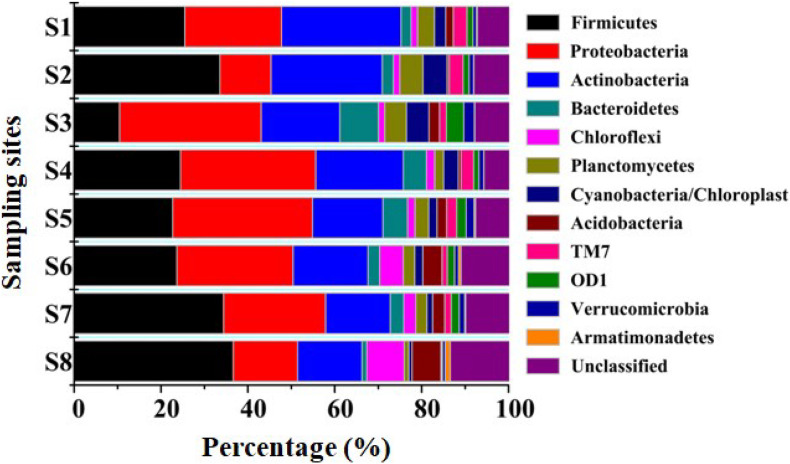
Relative abundances of different phyla in all sediment samples. The relative abundance was obtained by determined sequences vs. the total effective bacterial sequences with the help of RDP classifier at the threshold of 50%.

**FIGURE 3:**
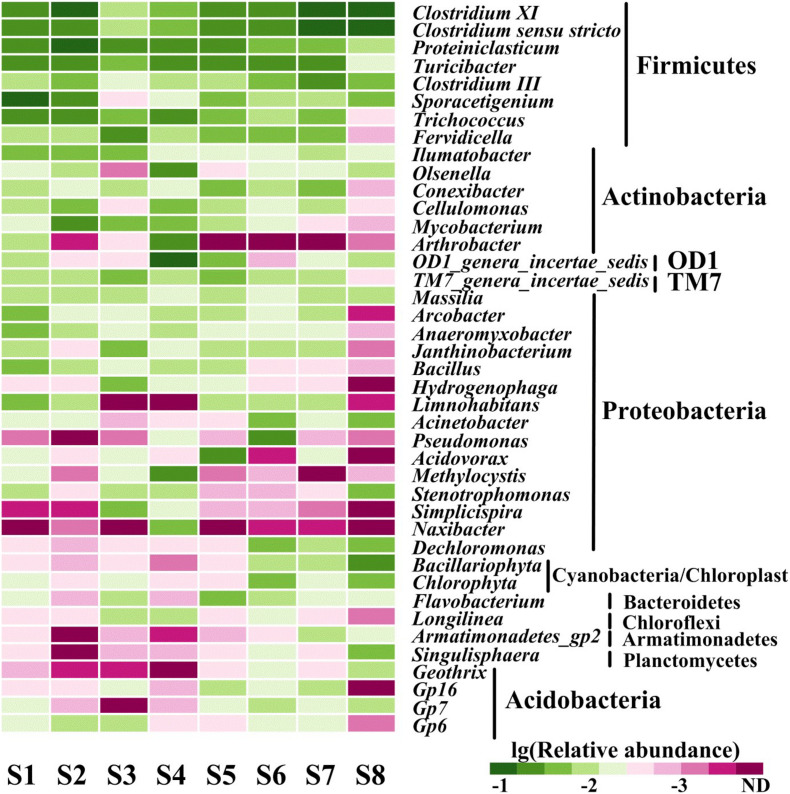
Heat map of genera (occurred at > 1% at least one sample) in all sediment samples. The relative abundance was obtained by determined sequences vs. the total effective bacterial sequences with the help of RDP classifier at the threshold of 50%.

Annotation against the RDP Classifier showed that the 7,395 effective bacterial sequences were assigned to different taxa levels (from genus to phylum) with a threshold of 50%. At the phyla level, Firmicutes (10.45–36.63%), Proteobacteria (11.76–32.59%) and Actinobacteria (14.81–27.45%) had the highest relative abundances. The other prevalent phyla primarily included Bacteroidetes (1.11–8.91%), Chloroflexi (1.42–8.61%), Planctomycetes (1.05–5.34%), Cyanobacteria/Chloroplast (0.72–5.56%), Acidobacteria (0.43–6.64%), TM7 (0.31–3.26%), OD1 (0.11–4%), Verrucomicrobia (0.58–2.24%) and Armatimonadetes (0.03–1.18%) (Figure 2). Proteobacteria, Firmicutes, Bacteroidetes, Acidobacteria and Actinobacteria were all found in anaerobic sediments that contained high concentrations of heavy metals (Sánchez-Andrea et al., 2011; Liu et al., 2018). Similarly, Firmicutes and Proteobacteria were both dominated in sediments contaminated with multiple heavy metals from the Xiangjiang and Beigang Rivers in China and in soils influenced by long-term chromium pollution (Desai et al., 2009; Zhu et al., 2013; Liu et al., 2018). This result was different from that of Proteobacteria and Acidobacteria, which contributed to a majority of the community composition in the less contaminated sediments (Sun et al., 2013). Moreover, the previous study showed that Deinococcus/Thermus phylum was positively associated with the presence of Cu and other heavy metals in the soil samples affected by the neutral mine drainage (Pereira et al., 2014). However, Deinococcus/Thermus was not detected in the present study with the high concentration of Cu. These differences might be due to the sediment chemical parameters and the concentrations of heavy metals (Sun et al., 2013; Pereira et al., 2014). The phylogenetic classification of sequences at the class level from the eight sediment samples is summarized in Supplementary Figure 1. In all the sampling sites, Clostridia (6.84–29.83%) and Actinobacteria (14.81–27.45%) were the first and the second dominant classes. The other dominant classes across all the sediments included Alphaproteobacteria (3.64–5.10%), Betaproteobacteria (3.27–17.78%), Gammaproteobacteria (1.27–7.61%), Deltaproteobacteria (0.91–6.30%), Bacilli (1.95–7.49%), Planctomycetacia (0.99–5.30%), and Chloroplast (0.68–4.90%). In a study conducted by Wang et al., Alphaproteobacteria, Gammaproteobacteria, and Deltaproteobacteria were also found by pyrosequencing to be the dominant classes in two typical intertidal sediments of Bohai Bay, China (Wang et al., 2013). As shown in Figure 3, a total of 41 genera had a relative abundance of >1% in one of the eight samples. Among the genera, *Clostridium XI* (1.35–9.47%), *Clostridium sensustricto* (1.09–11.47%), *Proteiniclasticum* (0.18–6.51%), *Cellulomonas* (0.64–4.59%), and *Mycobacterium* (1.39–7.73%) had relatively higher abundances in each sample.

### Linking Microbial Communities to Heavy Metals

A large proportion of the genera belonging to phylum of Firmicute generally dominated all the sampling sites, such as *Clostridium* XI and *Clostridium sensustricto*. Previous reports showed that *Clostridium* exhibited two tolerance mechanisms of heavy metals, including reductive precipitation and formation of heavy metal-protein complexes (Alexandrino et al., 2014; Maleke et al., 2019). These findings suggest *Clostridium* may improve the green technologies for bioremediation of heavy metals. In contrast, as the response to the heavy metals, the relative abundance of *Conexibacter*, *Cellulomonas* and *Mycobacterium* (Actinobacteria phylum) showed a tremendous decrease in the S8 sample with less contamination of various heavy metals. A similar trend occurred in the genera of *Arcobacter*, *Anaeromyxobacter*, *Janthinobacterium*, and *Bacillus* (Proteobacteria phylum), which was not consistent with the assumption that the genera would increase. Members of the *Cellulomonas* genus can effectively reduce Cr(VI) to Cr(III) fermentatively, indicating that they could play a potential role in the Cr(VI) remediation at Cr(VI) contaminated sites (Viamajala et al., 2007). Members of the genus *Arcobacter* are typically classified as nitrate-reducing and sulfide-oxidizing bacteria (Yeung et al., 2011). These two processes are usually associated with iron and manganese cycling that plays an important role in immobilization of heavy metals (Otth et al., 2005; Zhao and Dang, 2011; Yeung et al., 2011). The genus *Bacillus* has commonly been detected in soils, and its members (*Bacillus pumilus*, *Bacillus indicus*, *Bacillus Asus*, and *Bacillus clausii*) have exhibited high resistance against As, Cd, Co, Hg, Pb, and Se (Nithya et al., 2011).

**FIGURE 4:**
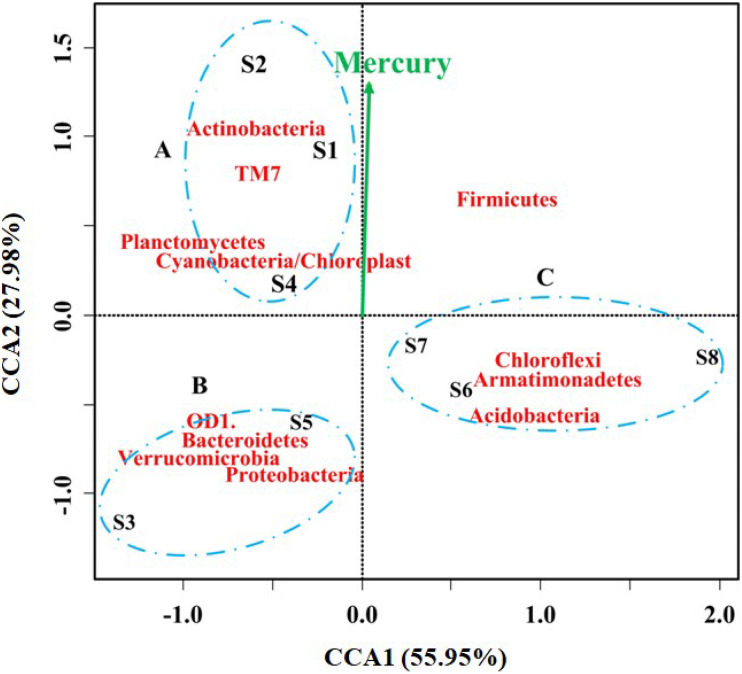
Correspondence Canonical Analysis (CCA) of the eight sediment samples at phylum level (55.95% of the scores variation could be explained by CCA1 and 27.98% by CCA2). Only the concentration of mercury was significantly correlated with the microbial community variation (*p* = 0.043, *r*^2^ = 0.748). The samples could be grouped into three clusters, cluster A included S1, S2 and S4, cluster B included S3 and S5, and cluster C included S6, S7, and S8.

Heavy metals have been reported to significantly affect the microbial diversity, activity, and biomass in the contaminated rivers (Chen J. et al., 2014; Liu et al., 2018; Li et al., 2020). For example, bioavailable Hg can damage microbial activities and inhibit enzymatic activities, resulting in selective pressure on microorganisms in Hg-polluted areas (Harris-Hellal et al., 2009; Mahbub et al., 2016, 2017). In this study, the microbial community profiles and concentrations of heavy metals were obtained from each sample to determine correlations between heavy metals and microbial populations using CCA. The strength of the effect of heavy metals on microbial community structure is reflected by the length of the arrow. As shown in Figure 4, a CCA ordination plot with the heavy metal concentrations is displayed for the three groups at the phylum level, and it can be seen that some heavy metals posed influence on the structure of microbial community. However, only the concentration of mercury was significantly correlated with the microbial community and species distribution (*p* = 0.043, *r*^2^ = 0.748). This result was consistent with the higher *E*_*i*_ in all the samples (Table 1). Additionally, the samples were generally divided into three clusters at the phylum level. Cluster A included S1, S2, and S4; cluster B included S3 and S5; and cluster C included S6, S7, and S8, which agreed with the contamination level of sampling locations and the river flow. The phyla of Actinobacteria, TM7, Planctomycetes and Cyanobacteria/Chloroplast dominated in cluster A, while the abundance of OD1, Bacteroidetes, Verrucomicrobia, and Proteobacteria was high in the sampling sites of S3 and S5. A previous study had revealed that Verrucomicrobia was positively correlated with increased mercury and methylmercury concentrations with low Hg concentration levels (Vishnivetskaya et al., 2011; Liu et al., 2014), but this was not consistent with the present result due to the high concentration of Hg in this study. Mahhub et al. reported a significant decrease in bacterial α diversity when the Hg concentration was up to 4.4 mg/kg (Mahbub et al., 2016). It is worth noting that the level of mercury that significantly (*p* < 0.05) explained the observed community variation was also shown at the level of class (Supplementary Figure 2) and order (Supplementary Figure 3). Moreover, the microbial community was similar among the less contaminated sites (S6, S7, and S8), where Chloroflexi, Armatimonadetes, and Acidobacteria dominated. Members of Firmicutes have been reported to possibly play a potential role in the transportation and deposition of trace metals in sediment conditions (Sauvain et al., 2014). These differences might be caused by the complex contaminations and other environmental parameters. As calculated, the sampling sites S1 and S4, S3 and S5, S6 and S7 had similar bacterial community structures at the phylum, class, and order levels, respectively, which were consistent with the changes in heavy metal concentrations in these locations and downstream along the river.

### Diversity and Abundances of As, Cd, and Cu Resistance Genes

**FIGURE 5:**
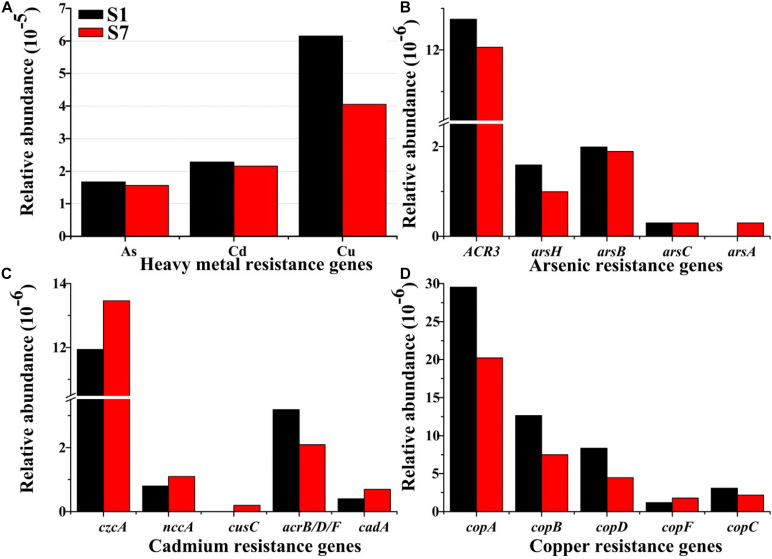
Exposure to heavy metal-contaminated effluent promotes HMRGs in bacterial communities in river sediment. The figure showed the relative abundance of **(A)** heavy metal resistance genes, **(B)** As resistance genes, **(C)** Cd resistance genes, and **(D)** Cu resistance genes. The relative abundance was calculated in relation to the total number of identified bacterial resistances genes.

Illumina high-throughput sequencing was conducted to investigate the diversity and abundance of the HMRGs in the selected samples of S1 and S7. After filtering and denoising, approximately 10 million clean reads (1.3 Gb) were finally generated for each sample. The results showed that the relative abundances of the Cu, Cd, and As resistance genes in sample S1 were all higher than those in S7 (Figure 5A, *p* < 0.05), suggesting that the relative abundance of HMRGs was well correlated with heavy metal concentrations (Jacquiod et al., 2018). The occurrence of heavy metals with high concentrations significant influenced the taxonomic and functional diversities of microbial communities in sediments (Romaniuk et al., 2018). Moreover, microorganisms demonstrated a variety of resistance mechanisms to adapt, accommodate, and live in the heavy metal-contaminated conditions, and the resistance mechanisms were mediated by autologous components and systems or the HMRGs (Rouch et al., 1995; Silver et al., 2001;Peltier et al., 2010). The ubiquitous, various and high-level heavy metals in the river sediments of the e-waste recycling area that exert a strong selective pressure on microorganisms, could induce HMRGs with long-term exposure, which would make HMRGs ubiquitous in microbial communities (Frostegård et al., 1996; Müller et al., 2001; Thomas et al., 2020). HMRGs considered as an emerging pollutant that can have an enormous impact on environmental safety, and they have increasingly become a major global human health threat (Peltier et al., 2010).

In a comparison of the As resistance, the database showed that 168 reads (0.017‰) and 157 reads (0.016‰) were identified as As resistance genes in samples S1 and S7, respectively (Figure 5A). The resistance genes of *ACR3*, *arsB*, *arsH* and *arsC* were present in all the samples, but *arsA* was only detected in sample S7. Various microbes showed resistance to As exposure and possessed the ars operon for As resistance. The ars operon consisted of three (*arsRBC*) to five (*arsRDABC*) genes organized into a single transcriptional unit (Kaur et al., 2011). In these As resistances, the role of *arsH* in As resistance remains contradictory since *arsH* from *Yersinia enterocolitica* confers resistance to both arsenite and arsenate, while *arsH* from *Acidothiobacillus ferrooxidans* did not appear to confer As resistance (Neyt et al., 1997; Butcher et al., 2000). Among the identified As resistance genes, the *ACR3* gene had the highest relative abundance (Figure 5B) and was found in *Stenotrophomonas maltophilia*, *Starkeya novella*, *Geobacter sp*., and *Deferribacter desulfuricans* (Supplementary Table 5). The database showed that the *ArsH* gene was widely distributed in *Stenotrophomonas maltophilia*, *Yersinia enterocolitica* and *Acinetobacter sp*. (Supplementary Table 5). Efflux is one of the most common mechanisms that microorganisms utilize to obtain heavy metal resistance due to the environmental pervasiveness of As (Alvarez et al., 2017). The previous reports showed that the As efflux protein, *ACR3*, was widespread (Fu et al., 2009), which was consistent with the highest relative abundance of *ACR3* in the sediments.

Similarly, 229 reads (0.023‰) and 216 reads (0.022‰) identified from samples S1 to S7, respectively, were responsible for Cd resistance (Figure 5A). Cd resistance genes including *czcA*, *nccA*, *acrB/D/F*, and *cadA* were present in both samples, while *cusC* was detected only in sample S7. Among the detected genes, the *czcA* gene had the highest abundance and had a broad range of host species including *Herminiimonas arsenicoxydans*, *Acinetobacter baumannii*, *Methylocystis* sp., Ralstonia sp., and *Oxalobacteraceae bacterium* (Figure 5C). The *czcA* gene belongs to the efflux system *czc* and can pump Co(II), Zn(II), and Cd(II) from cells by encoding the cobalt-zinc-cadmium resistance protein (Trevors et al., 1986; Romaniuk et al., 2018). Roosa et al. found an interesting phenomenon that the *czcA* gene might be co-selected by other non-target metals such as Cu and As (Roosa et al., 2014). Moreover, the *czcA* protein encoded by the *czc* operon is a member of the RND family, which modulates low level resistance to Co(II), Zn(II), and Cd(II) (Rensing et al., 1997; Roosa et al., 2014). The second highest abundance of the Cd resistance gene (*cad*) was a two-component operon that consisted of two genes designated as *cadA* and *cadD* (Zhang et al., 2015). The *cadA* gene encods a Cd^2+^/ATPase protein transporter to accommodate and counteract heavy metals stress, while *cadD* genes enhance Cd resistance (Hsieh et al., 2010; Zheng et al., 2019).

Furthermore, alignment against the Cu resistance database showed that the Cu resistance genes were more abundant than the As and Cd resistance genes in each sediment sample (Figure 5A). This has been due to the highest concentration of Cu in all the sediment samples (Supplementary Table 3). Previous report showed a positive correlation between the level of *copA* and Cu in paddy soils exposed to 1-year of Cu contamination (Li et al., 2012). A total of 618 reads (0.061‰) and 406 reads (0.040‰) were assigned to the Cu resistance genes, including *copA*, *copB*, *copC*, *copD*, and *copF* (Figure 5D). These results also showed that the multi Cu oxidase gene, *copA*, that determines the uptake P-type ATPase, dominated in the microbial communities, and this was followed by *copB* coding for the efflux P-type ATPase. These two genes were found in a single operon and are currently best understood as a Cu resistance and transport system (Teixeira et al., 2008). This *cop* system was regulated in response to Cu-starvation when the *copA* uptake ATPase was needed, or to Cu-excess when the *copB* efflux ATPase was needed (Behlau et al., 2011; Chen et al., 2019). The primary bacterial hosts of the Cu resistance genes (primary *copA*) were found to be *Acinetobacter radioresistens*, *Methyloversatilis universalis*, *Xanthomonas vesicatoria*, *Pseudoxanthomonas spadix*, and *Oxalobacteraceae bacterium* (Supplementary Table 5).

## Concusion and Implications

In this study, our results have shed light on the diversity and composition of the microbial communities in river sediments seriously contaminated by e-waste recycling. 454 pyrosequencing showed that Firmicutes, Proteobacteria, and Actinobacteria dominated the sediment microbial assemblages followed by Bacteroidetes and Chloroflexi. Specifically, the abundance of Firmicute increased along with the decreased level of contaminants. Inversely, there was a gradual decline trend in the abundance of Actinobacteria. Statistical analysis revealed that the concentration of mercury was significantly correlated with the microbial community and species distribution, which agreed with an analysis of the potential ecological risk index. With metagenomic analysis, the relative abundance of heavy metal resistance genes was related with the contamination level and the exposure time. For instance, the abundances of the *arsB* and *ACR3* genes correlated positively with the As(III) concentration in a wasteland soil (Poirel et al., 2013). However, the direct correlation between the abundance and concentration of heavy metals is difficult to quantify due to complex contaminated conditions and the bioaccessibility of heavy metals (Ye et al., 2016; Liu et al., 2019). Some of the identified HMRGs might be inactive, or the presence of a mutation or genetic incongruity could exist in an e-waste recycling area (Dziewit et al., 2015; Romaniuk et al., 2018). To depict a more-detailed picture among microbial communities, functional microorganisms and HMRGs in contaminated sediments, other shotgun omics technologies need to carry out to explore all microbial genomes, proteomes, and complete transcriptomes. Nevertheless, characterization of microbial communities and HMRGs in this study can provide more information for bio-remediation in contaminated sediments or act as eco-indicators for eco-toxicological research.

## Data Availability Statement

The datasets presented in this study can be found in online repositories. The names of the repository/repositories and accession number(s) can be found below: (National Center for Biotechnology Information AND SRA221687).

## Author Contributions

CL and XZ conceived and designed the experiments. SL and HT performed the experiments. SJ and MC analyzed the data. All authors contributed to the article and approved the submitted version.

## Conflict of Interest

The authors declare that the research was conducted in the absence of any commercial or financial relationships that could be construed as a potential conflict of interest.

## Footnotes

1 https://www.ncbi.nlm.nih.gov/

2 http://www.mothur.org
