# Supplementary material for: Heavy Metal Tolerance Genes Associated With Contaminated Sediments From an E-Waste Recycling River in Southern China
Source: Front Microbiol. 2021 May 13;12:665090. doi: 10.3389/fmicb.2021.665090 (PMC8155521; doi:10.3389/fmicb.2021.665090)
Supplement: Supplementary file 1 [file Data_Sheet_1.docx]

***Supplementary materials***

**Heavy metal tolerance genes associated with contaminated sediments from** **an e-waste recycling river in southern China**

Shengqiao Long ^1, 2#^, Hui Tong ^3#^, Xuxiang Zhang ^4^, Shuyu Jia ^4^, Manjia Chen ^3^, Chengshuai Liu ^1, 3*^

^1^ *State Key Laboratory of Environmental Geochemistry, Institute of Geochemistry, Chinese Academy of Sciences, Guiyang 550081, China*

^2^ *University of Chinese Academy of Sciences, Beijing 100049, PR China*

^3^ *National-Regional Joint Engineering Research Center for Soil Pollution Control and Remediation in South China, Guangdong Key Laboratory of Integrated Agro-environmental Pollution Control and Management, Guangdong Institute of Eco-environmental Science & Technology, Guangdong Academy of Sciences, Guangzhou 510650, China*

^4^ *State Key Laboratory of Pollution Control and Resource Reuse, School of the Environment, Nanjing University, Nanjing 210023, China*

^#^ These authors contributed equally to this work.

**CORRESPONDENCE:** Dr. Chengshuai Liu (liuchengshuai@vip.gyig.ac.cn)





**Figure S1.** Heat map of classes (occurred at > 1% at least one sample) in the 8 sediment samples. The relative abundance was obtained by determined sequences versus the total effective bacterial sequences with the help of RDP classifier at the threshold of 50%.

**
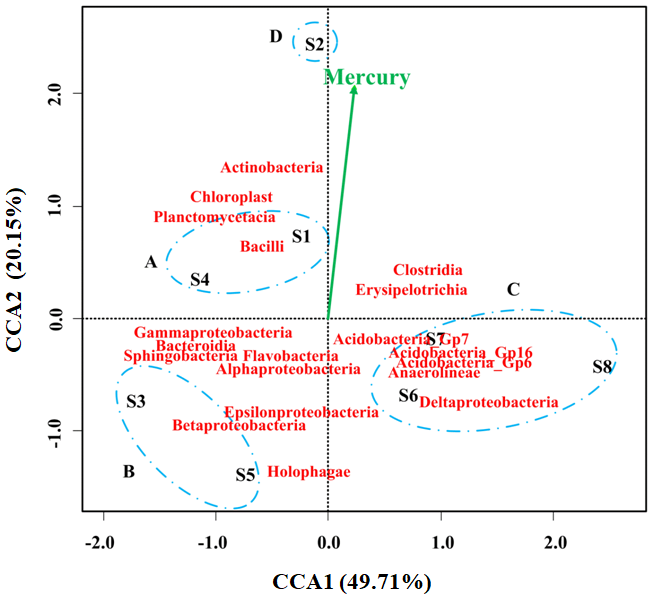
**

**Figure S2**. CCA of the eight sediment samples at class level (49.71% of the scores variation could be explained by CCA1 and 20.15% CCA2). Only the concentration of mercury was significantly correlated with the microbial community variation (*p* = 0.047, r^2^ = 0.705). The samples could be grouped into four clusters, cluster A included S1 and S4, cluster B included S3 and S5, cluster C included S6, S7 and S8, and S2 independently formed the cluster D.


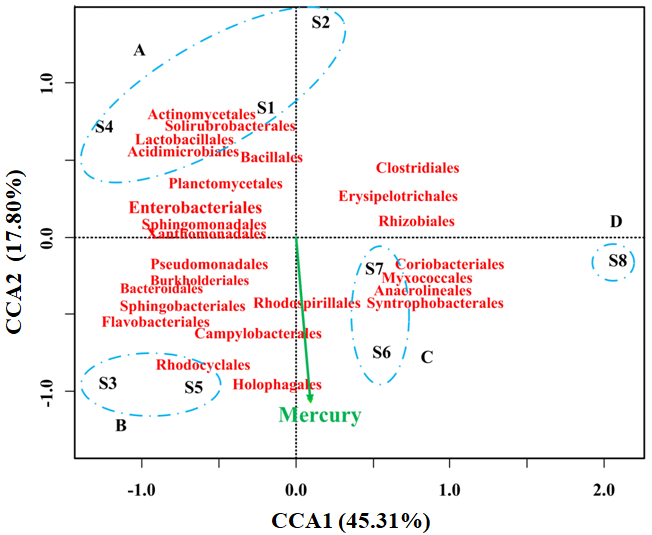


**Figure S3**. CCA of the eight sediment samples at class level (45.31% of the scores variation could be explained by CCA1 and 17.80% by CCA2). Only the concentration of mercury was significantly correlated with the microbial community variation (*p* = 0.023, r^2^ = 0.751). The samples could be grouped into four clusters, cluster A included S1, S2 and S4, cluster B included S3 and S5, cluster C included S6 and S7, and S8 sample independently formed the cluster D.

**Table S1** The longitude and latitude for all samples in Figure 1

| Sample | Latitude (N) | Longitude (E) |
| --- | --- | --- |
| S1 | 23°35′27.96″ | 113°2′5.06″ |
| S2 | 23°35′25.42″ | 113°2′6.69″ |
| S3 | 23°35′21.60″ | 113°2′6.42″ |
| S4 | 23°35′22.16″ | 113°2′10.42″ |
| S5 | 23°35′16.94″ | 113°2′10.59″ |
| S6 | 23°35′12.19″ | 113°2′9.87″ |
| S7 | 23°35′8.27″ | 113°2′10.14″ |
| S8 | 23°35′4.88″ | 113°2′8.53″ |

**Table S2** Judged standard of contamination degree by potential ecological risk index

| *E_i_* | RI | Potential ecological risk level |
| --- | --- | --- |
| *E_i_* < 40 | RI < 150 | Low |
| 40 ≤ *E_i_* < 80 | 150 ≤ RI < 300 | Moderate |
| 80 ≤ *E_i_* < 160 | 300 ≤ RI < 600 | Severe |
| 160 ≤ *E_i_* < 320 | RI ≥ 600 | Serious |
| *E_i_* ≥ 320 |  | Serious |

**Table S3.** Concentrations of heavy metal in the sediments (μg/g)

| Names | As | Cd | Cu | Hg | Ni | Cr | Pb | Zn |
| --- | --- | --- | --- | --- | --- | --- | --- | --- |
| Standard value^1^ | 17.39 | 0.82 | 20.17 | 0.26 | 42.44 | 88.01 | 101.24 | 256.67 |
| Background of China^2^ | 11.2 | 0.037 | 20.4 | 0.053 | 18.2 | 58.9 | 40.1 | 51.7 |
| S1 | 307.30 | 53.56 | 101128.50 | 7.75 | 215.14 | 814.95 | 2357.31 | 4467.26 |
| S2 | 90.11 | 33.74 | 8914.81 | 14.99 | 177.61 | 483.32 | 1796.57 | 2021.27 |
| S3 | 124.61 | 20.47 | 5495.65 | 3.97 | 131.02 | 140.70 | 1403.24 | 2219.05 |
| S4 | 104.65 | 47.91 | 7844.70 | 5.56 | 180.09 | 533.94 | 2484.34 | 3798.69 |
| S5 | 60.48 | 18.61 | 5692.06 | 5.40 | 108.65 | 319.03 | 1233.61 | 2392.23 |
| S6 | 33.43 | 11.16 | 4431.04 | 5.67 | 108.88 | 290.13 | 1267.31 | 2132.78 |
| S7 | 45.90 | 29.50 | 6917.27 | 10.40 | 156.26 | 443.05 | 1554.65 | 4296.30 |
| S8 | 35.03 | 21.91 | 4534.79 | 3.35 | 120.02 | 111.26 | 692.31 | 2442.53 |

^1^Environmental Quality Standard for Soils regulated by Environmental Protection Agency of China; ^2^Data from China National Environmental Monitoring Center.

**Table S4.** Microbial diversity of the sediment samples analyzed based on pyrosequencing of 16S rRNA gene

| Sample | Number of OTUs^1^ | Chao1^2^ | ACE^3^ | Shannon | Simpson |
| --- | --- | --- | --- | --- | --- |
| S1 | 2092 | 4131.76 | 3919.51 | 6.73 | 0.003011 |
| S2 | 1978 | 3896.37 | 3672.75 | 6.61 | 0.003792 |
| S3 | 2528 | 5125.88 | 4836.51 | 7.11 | 0.001819 |
| S4 | 1908 | 3940.55 | 3608.68 | 6.48 | 0.00472 |
| S5 | 2429 | 5214.16 | 4762.74 | 6.99 | 0.002253 |
| S6 | 2494 | 4931.92 | 4708.06 | 7.04 | 0.002499 |
| S7 | 2474 | 5043.33 | 4701.75 | 6.95 | 0.003170 |
| S8 | 2405 | 4533.39 | 4346.74 | 6.99 | 0.002405 |

^1^OTUs, operational taxonomic units; ^2^Chao, Chao estimator; ^3^ACE, the abundance-based coverage estimator.

**Table S5.** The information of identified heavy mental resistance genes in this study.

| Sample | Accession No. | Number of reads | Gene | Function | Bacterial host |
| --- | --- | --- | --- | --- | --- |
| S1 | YP_003693513.1 | 11 | *ACR3* | Arsenic membrane transporter | *Starkeya novella* |
|  | AAY85171.1 | 10 | *arsB* | Arsenite transmembrane pump | *Leptospirillum ferriphilum* |
|  | YP_003021684.1 | 9 | *ACR3* | Arsenic membrane transporter | *Geobacter* sp. |
|  | YP_004013809.1 | 9 | *ACR3* | Arsenic membrane transporter | *Rhodomicrobium vannielii* |
|  | YP_111445.1 | 9 | *ACR3* | Arsenic membrane transporter | *Burkholderia pseudomallei* |
|  | YP_002136352.1 | 8 | *ACR3* | Arsenic membrane transporter | *Anaeromyxobacter* sp. |
|  | YP_003496669.1 | 7 | *ACR3* | Arsenic membrane transporter | *Deferribacter desulfuricans* |
|  | NP_902110.1 | 7 | *ACR3* | Arsenic resistance membrane protein | *Chromobacterium violaceum* |
|  | YP_001892366.1 | 6 | *ACR3* | Arsenic membrane transporter | *Ralstonia pickettii* |
|  | YP_004099318.1 | 5 | *ACR3* | Arsenic membrane transporter | *Intrasporangium calvum* |
|  | ADZ05767.1 | 5 | *arsB* | Arsenical resistance protein | *Acinetobacter baumannii* |
|  | ABF22612.1 | 5 | *arsB* | Arsenite membrane pump | *Ochrobactrum tritici* |
|  | YP_002892220.1 | 4 | *ACR3* | Arsenic membrane transporter | *Tolumonas auensis* |
|  | ZP_08235443.1 | 4 | *ACR3* | Arsenic membrane transporter | *Streptomyces griseus* |
|  | YP_001715386.1 | 4 | *arsH* | Arsenical resistance protein | *Acinetobacter baumannii* |
|  | ZP_03824810.1 | 4 | *arsH* | Arsenical resistance protein | *Acinetobacter* sp. |
|  | YP_703329.1 | 3 | *ACR3* | Arsenic membrane transporter | *Rhodococcus jostii* |
|  | YP_001132927.1 | 3 | *ACR3* | Arsenic membrane transporter | *Mycobacterium gilvum* |
|  | YP_001972211.1 | 3 | *ACR3* | Arsenic membrane transporter | *Stenotrophomonas maltophilia* |
|  | YP_003608693.1 | 3 | *ACR3* | Arsenic membrane transporter | *Burkholderia* sp. |
|  | ZP_08288615.1 | 3 | *ACR3* | Arsenic membrane transporter | *Streptomyces griseoaurantiacus* |
|  | YP_641682.1 | 2 | *ACR3* | Arsenic membrane transporter | *Mycobacterium* sp. |
|  | YP_829713.1 | 2 | *ACR3* | Arsenic membrane transporter | *Arthrobacter* sp. |
|  | YP_001893862.1 | 2 | *ACR3* | Arsenic membrane transporter | *Burkholderia phytofirmans* |
|  | YP_002355501.1 | 2 | *ACR3* | Arsenic membrane transporter | *Thauera* sp. |
|  | YP_002988705.1 | 2 | *ACR3* | Arsenic membrane transporter | *Dickeya dadantii* |
|  | YP_004246759.1 | 2 | *ACR3* | Arsenic membrane transporter | *Sphaerochaeta globus* str. |
|  | ACL68351.1 | 2 | *arsC* | Arsenate reductase-like protein | *Bacillus* sp. |
|  | XP_001182180.1 | 2 | *arsH* | Arsenic resistance protein | *Strongylocentrotus purpuratus* |
|  | YP_003977145.1 | 2 | *arsH* | Arsenical resistance protein | *Achromobacter xylosoxidans* |
|  | YP_001069735.1 | 1 | *ACR3* | Arsenic membrane transporter | *Mycobacterium* sp. |
|  | ZP_01739410.1 | 1 | *ACR3* | Arsenic membrane transporter | *Marinobacter* sp. |
|  | YP_001301411.1 | 1 | *ACR3* | Arsenic membrane transporter | *Bacteroides vulgatus* |
|  | YP_002422897.1 | 1 | *ACR3* | Arsenic membrane transporter | *Methylobacterium chloromethanicum* |
|  | YP_002457706.1 | 1 | *ACR3* | Arsenic membrane transporter | *Desulfitobacterium hafniense* |
|  | YP_003272002.1 | 1 | *ACR3* | Arsenic membrane transporter | *Gordonia bronchialis* |
|  | YP_003273802.1 | 1 | *ACR3* | Arsenic membrane transporter | *Gordonia bronchialis* |
|  | YP_003340106.1 | 1 | *ACR3* | Arsenic membrane transporter | *Streptosporangium roseum* |
|  | YP_003392399.1 | 1 | *ACR3* | Arsenic membrane transporter | *Conexibacter woesei* |
|  | NP_809027.1 | 1 | *ACR3* | Arsenic membrane transporter | *Bacteroides thetaiotaomicron* |
|  | CBH20088.1 | 1 | *ACR3* | Arsenic membrane transporter | *Desulfovibrio* sp. |
|  | YP_003647181.1 | 1 | *ACR3* | Arsenic membrane transporter | *Tsukamurella paurometabola* |
|  | YP_003756459.1 | 1 | *ACR3* | Arsenic membrane transporter | *Hyphomicrobium denitrificans* |
|  | NP_856322.1 | 1 | *ACR3* | Arsenic-transport integral membrane protein | *Mycobacterium bovis* |
|  | YP_004270610.1 | 1 | *ACR3* | Arsenic membrane transporter | *Planctomyces brasiliensis* |
|  | YP_004264406.1 | 1 | *ACR3* | Arsenic membrane transporter | *Syntrophobotulus glycolicus* |
|  | YP_004319955.1 | 1 | *ACR3* | Arsenic membrane transporter | *Sphingobacterium* sp. |
|  | YP_004806297.1 | 1 | *ACR3* | Arsenic membrane transporter | *Streptomyces* sp. |
|  | YP_122074.1 | 1 | *ACR3* | Arsenic membrane transporter | *Nocardia farcinica* |
|  | ZP_00997259.1 | 1 | *ACR3* | Putative arsenic resistance transporter | *Janibacter* sp. |
|  | YP_462982.1 | 1 | *ACR3* | Arsenic membrane transporter | *Syntrophus aciditrophicus* |
|  | ABF48394.1 | 1 | *ACR3* | Arsenite efflux pump | *Ochrobactrum tritici* |
|  | ABF48393.1 | 1 | *arsC* | Arsenate reductase | *Ochrobactrum tritici* |
|  | ZP_07045193.1 | 1 | *arsH* | Arsenical resistance protein | *Comamonas testosteroni* |
|  | ZP_08276051.1 | 1 | *arsH* | Arsenical resistance protein | *Oxalobacteraceae bacterium* |
|  | YP_004792856.1 | 1 | *arsH* | Arsenical resistance protein | *Stenotrophomonas maltophilia* |
|  | ZP_08952179.1 | 1 | *arsH* | Arsenical resistance protein | *Rhodanobacter* sp. |
|  | ZP_08803718.1 | 1 | Others | Tyrosine phosphatase | *Streptomyces zinciresistens* |
|  | YP_004042151.1 | 27 | *acrB/D/F* | Heavy metal efflux pump | *Paludibacter propionicigenes* |
|  | YP_001099897.1 | 13 | *czcA* | Cobalt-zinc-cadmium resistance protein | *Herminiimonas arsenicoxydans* |
|  | ZP_08441696.1 | 11 | *czcA* | Cobalt-zinc-cadmium resistance protein | *Acinetobacter baumannii* |
|  | ZP_08072272.1 | 10 | *czcA* | Cobalt-zinc-cadmium resistance protein | *Methylocystis* sp. |
|  | EFR90519.1 | 9 | *cadD* | Cadmium resistance transporter | *Listeria innocua* |
|  | ZP_07673472.1 | 8 | *czcA* | Cobalt-zinc-cadmium resistance protein | *Ralstonia* sp. |
|  | ZP_08275386.1 | 8 | *czcA* | Cobalt-zinc-cadmium resistance protein | *Oxalobacteraceae bacterium* |
|  | ZP_08250611.1 | 7 | *cadD* | Cadmium resistance family protein | *Dialister micraerophilus* |
|  | NCCA_ALCXX | 7 | *nccA* | Nickel-cobalt-cadmium resistance protein nccA | *Achromobacter xylosoxidans* |
|  | YP_001974238.1 | 5 | *czcA* | Cobalt-zinc-cadmium resistance protein | *Stenotrophomonas maltophilia* |
|  | ZP_08629997.1 | 5 | *czcA* | Cobalt-zinc-cadmium resistance protein | *Bradyrhizobiaceae bacterium* |
|  | ZP_06061604.1 | 5 | Others | Cobalt-zinc-cadmium efflux pump | *Acinetobacter johnsonii* |
|  | YP_003985890.1 | 4 | *cadD* | Cadmium resistance transporter | *Gardnerella vaginalis* |
|  | YP_004599354.1 | 4 | *cadD* | Cadmium resistance transporter | *Cellvibrio gilvus* |
|  | ZP_07664505.1 | 4 | *cadD* | Cadmium resistance transporter | *Atopobium vaginae* |
|  | YP_931731.1 | 4 | *czcA* | Cobalt-zinc-cadmium resistance protein | *Azoarcus* sp. |
|  | ZP_06886456.1 | 4 | *czcA* | Cobalt-zinc-cadmium resistance protein | *Methylosinus trichosporium* |
|  | YP_003777808.1 | 4 | *czcA* | Cobalt-zinc-cadmium resistance protein | *Herbaspirillum seropedicae* |
|  | YP_145595.1 | 4 | *czcA* | Cation efflux system transmembrane protein | *Cupriavidus metallidurans* |
|  | ZP_06501487.1 | 3 | *cadD* | Cadmium resistance transporter | *Micrococcus luteus* |
|  | YP_931772.1 | 3 | *czcA* | Cobalt-zinc-cadmium resistance protein | *Azoarcus* sp. |
|  | ZP_03543140.1 | 3 | *czcA* | Cobalt-zinc-cadmium resistance protein | *Comamonas testosteroni* |
|  | YP_003280424.1 | 3 | *czcA* | Cobalt-zinc-cadmium resistance protein | *Comamonas testosteroni* |
|  | YP_003629615.1 | 3 | *czcA* | Cobalt-zinc-cadmium resistance protein | *Planctomyces limnophilus* |
|  | ZP_08276460.1 | 3 | *czcA* | Cobalt-zinc-cadmium resistance protein | *Oxalobacteraceae bacterium* |
|  | ZP_08506022.1 | 3 | *czcA* | Cobalt-zinc-cadmium resistance protein | *Methyloversatilis universalis* |
|  | ZP_00945440.1 | 3 | *czcA* | Cobalt-zinc-cadmium resistance protein | *Ralstonia solanacearum* |
|  | NP_421193.1 | 2 | *acrB/D/F* | Heavy metal efflux pump | *Caulobacter crescentus* |
|  | YP_004451316.1 | 2 | *acrB/D/F* | Heavy metal efflux pump | *Haliscomenobacter hydrossis* |
|  | AF333961_2 | 2 | *cadA* | Cadmium transporting ATPase | *Pseudomonas putida* |
|  | YP_003635466.1 | 2 | *cadD* | Cadmium resistance transporter | *Cellulomonas flavigena* |
|  | YP_954439.1 | 2 | *cadD* | Cadmium resistance transporter | *Mycobacterium vanbaalenii* |
|  | YP_001134476.1 | 2 | *cadD* | Cadmium resistance transporter | *Mycobacterium gilvum* |
|  | ZP_03305283.1 | 2 | *cadD* | Cadmium resistance transporter | *Anaerococcus hydrogenalis* |
|  | ZP_07819498.1 | 2 | *cadD* | Cadmium resistance transporter | *Eremococcus coleocola* |
|  | ZP_06068071.1 | 2 | *czcA* | Cobalt-zinc-cadmium resistance protein | *Acinetobacter junii* |
|  | ZP_06729092.1 | 2 | *czcA* | Cobalt-zinc-cadmium resistance protein | *Acinetobacter haemolyticus* |
|  | YP_004258364.1 | 2 | *czcA* | Cobalt-zinc-cadmium resistance protein | *Bacteroides salanitronis* |
|  | ZP_08272425.1 | 2 | *czcA* | Cobalt-zinc-cadmium resistance protein | *gamma proteobacterium* |
|  | EHC73749.1 | 2 | *czcA* | Cobalt-zinc-cadmium resistance protein | *Salmonella enterica* subsp. |
|  | YP_002233336.1 | 2 | *czcA* | Cobalt-zinc-cadmium resistance transporter protein | *Burkholderia cenocepacia* |
|  | YP_002553649.1 | 2 | *czcB* | Cobalt-zinc-cadmium resistance protein | *Acidovorax ebreus* |
|  | YP_145594.1 | 2 | *czcB* | Cobalt-zinc-cadmium resistance protein | *Cupriavidus metallidurans* |
|  | YP_637683.1 | 2 | *MMT1* | Cation efflux protein | *Mycobacterium* sp. |
|  | YP_004046468.1 | 2 | *MMT1* | Cation efflux protein | *Riemerella anatipestifer* |
|  | ZP_08072271.1 | 2 | Others | efflux transporter | *Methylocystis* sp. |
|  | YP_301693.1 | 2 | Others | Cadmium resistance protein | *Staphylococcus saprophyticus* subsp. |
|  | ZP_08457412.1 | 1 | *acrB/D/F* | Heavy metal efflux pump | *Bacteroides coprosuis* |
|  | ABR09922.1 | 1 | *cadA* | Cadmium resistance ATPase | *Bacillus* sp. |
|  | NP_898745.1 | 1 | *cadA* | Putative cadmium resistance protein | *Rhodococcus erythropolis* |
|  | ZP_07091299.1 | 1 | *cadD* | Cadmium resistance transporter | *Corynebacterium genitalium* |
|  | ZP_02156302.1 | 1 | *czcA* | Cobalt-zinc-cadmium resistance protein | *Shewanella benthica* |
|  | ZP_03821708.1 | 1 | *czcA* | Cobalt-zinc-cadmium resistance protein | *Acinetobacter* sp. |
|  | YP_002870366.1 | 1 | *czcA* | Cobalt-zinc-cadmium resistance protein | *Pseudomonas fluorescens* |
|  | YP_003754577.1 | 1 | *czcA* | Cobalt-zinc-cadmium resistance protein | *Hyphomicrobium denitrificans* |
|  | YP_003912928.1 | 1 | *czcA* | Cobalt-zinc-cadmium resistance protein | *Ferrimonas balearica* |
|  | EFV84518.1 | 1 | *czcA* | Cobalt-zinc-cadmium resistance protein | *Achromobacter xylosoxidans* |
|  | NP_869864.1 | 1 | *czcA* | Cobalt-zinc-cadmium resistance protein | *Rhodopirellula baltica* |
|  | YP_004866524.1 | 1 | *czcA* | Cobalt-zinc-cadmium resistance protein | *Micavibrio aeruginosavorus* |
|  | EHC73733.1 | 1 | *czcA* | Cobalt-zinc-cadmium resistance protein | *Salmonella enterica* subsp. |
|  | YP_262295.1 | 1 | *czcA* | Cobalt-zinc-cadmium resistance protein | *Pseudomonas protegens* |
|  | YP_001972238.1 | 1 | *czcA* | Cobalt-zinc-cadmium resistance protein | *Stenotrophomonas maltophilia* |
|  | ZP_08262060.1 | 1 | *nccA* | Nickel-cobalt-cadmium resistance protein nccA | *Asticcacaulis biprosthecum* |
|  | ZP_07362750.1 | 1 | *zntA* | Cation transport ATPase | *Staphylococcus aureus* subsp. |
|  | EFU16574.1 | 1 | Others | Cadmium resistance transporter | *Enterococcus faecalis* |
|  | ZP_09024038.1 | 1 | Others | Cadmium resistance transporter | *Propionibacterium* sp. |
|  | ZP_05665620.1 | 1 | Others | Cadmium resistance transporter | *Enterococcus faecium* |
|  | YP_003846723.1 | 1 | Others | Outer membrane efflux protein | *Gallionella capsiferriformans* |
|  | YP_004096061.1 | 1 | Others | ATPase P | *Bacillus cellulosilyticus* |
|  | ZP_05361756.1 | 102 | *copA* | Multicopper oxidase type 3 | *Acinetobacter radioresistens* |
|  | ZP_05361757.1 | 47 | *copB* | Copper resistance protein B precursor | *Acinetobacter radioresistens* |
|  | ZP_08506054.1 | 40 | *copA* | Multicopper oxidase type 3 | *Methyloversatilis universalis* |
|  | ZP_07567309.1 | 39 | *copB* | Copper resistance protein B precursor | *Enterococcus faecalis* |
|  | ZP_06073977.1 | 32 | *copD* | Copper resistance protein | *Acinetobacter radioresistens* |
|  | EGO37246.1 | 18 | *copD* | Copper resistance protein | *Mycobacterium avium* |
|  | YP_004929441.1 | 12 | *copA* | Multicopper oxidase type 3 | *Pseudoxanthomonas spadix* |
|  | ZP_05361746.1 | 12 | *copC* | Copper resistance protein | *Acinetobacter radioresistens* |
|  | ZP_06064061.1 | 11 | *copD* | Putative copper export protein | *Acinetobacter johnsonii* |
|  | CAC07984.1 | 11 | *copF* | Cu-ATPase | *Cupriavidus metallidurans* |
|  | YP_004346534.1 | 10 | *copA* | Multicopper oxidase type 3 | *Fluviicola taffensis* |
|  | ZP_02185059.1 | 10 | Others | Cation-transporting ATPase | *Carnobacterium* sp. |
|  | ZP_08433026.1 | 7 | *copA* | Multicopper oxidase type 3 | *Acinetobacter baumannii* |
|  | ZP_08435630.1 | 7 | *copA* | Multicopper oxidase type 3 | *Acinetobacter baumannii* |
|  | YP_124656.1 | 7 | *copA* | Multicopper oxidase type 3 | *Legionella pneumophila* |
|  | ZP_06726395.1 | 6 | *copA* | Multicopper oxidase type 3 | *Acinetobacter haemolyticus* |
|  | YP_004046465.1 | 6 | *copA* | Multicopper oxidase type 3 | *Riemerella anatipestifer* |
|  | ZP_07043165.1 | 6 | *copB* | Copper resistance protein B precursor | *Comamonas testosteroni* |
|  | YP_001899418.1 | 5 | *copA* | Copper resistance protein | *Ralstonia pickettii* |
|  | ZP_06062488.1 | 5 | *copA* | Multicopper oxidase type 3 | *Acinetobacter johnsonii* |
|  | ZP_07089169.1 | 5 | *copA* | Multicopper oxidase type 3 | *Chryseobacterium gleum* |
|  | YP_004929428.1 | 5 | *copD* | Copper resistance protein | *Pseudoxanthomonas spadix* |
|  | YP_001561483.1 | 5 | Others | Heavy metal sensor signal transduction histidine kinase | *Delftia acidovorans* |
|  | YP_001845311.1 | 4 | *copA* | Multicopper oxidase type 3 | *Acinetobacter baumannii* |
|  | ZP_07082318.1 | 4 | *copA* | Multicopper oxidase type 3 | *Sphingobacterium spiritivorum* |
|  | ZP_08268022.1 | 4 | *copA* | Multicopper oxidase type 3 | *Brevundimonas diminuta* |
|  | YP_004682352.1 | 4 | *copA* | Multicopper oxidase type 3 | *Cupriavidus necator* |
|  | YP_004843209.1 | 4 | *copA* | Multicopper oxidase type 3 | *Flavobacterium branchiophilum* |
|  | ZP_01303652.1 | 4 | *copA* | Multicopper oxidase type 3 | *Sphingomonas* sp. |
|  | YP_001899286.1 | 4 | *copC* | Copper resistance protein | *Ralstonia pickettii* |
|  | ZP_06726389.1 | 4 | *copD* | Putative copper export protein | *Acinetobacter haemolyticus* |
|  | NP_301251.1 | 4 | Others | Heavy metal sensor signal transduction histidine kinase | *Mycobacterium leprae* |
|  | ZP_09008857.1 | 4 | Others | heavy metal translocating P-type ATPase | *Mycobacterium rhodesiae* |
|  | YP_746278.1 | 3 | *copA* | Multicopper oxidase type 3 | *Nitrosomonas eutropha* |
|  | YP_001419480.1 | 3 | *copA* | Multicopper oxidase type 3 | *Xanthobacter autotrophicus* |
|  | ZP_06066684.1 | 3 | *copA* | Multicopper oxidase type 3 | *Acinetobacter junii* |
|  | ZP_06686120.1 | 3 | *copA* | Multicopper oxidase type 3 | *Achromobacter piechaudii* |
|  | YP_003618340.1 | 3 | *copA* | Multicopper oxidase type 3 | *Legionella pneumophila* |
|  | YP_002973805.1 | 3 | *copB* | Copper resistance protein B precursor | *Ralstonia pickettii* |
|  | ZP_08506053.1 | 3 | *copB* | Copper resistance protein B precursor | *Methyloversatilis universalis* |
|  | CCC56739.1 | 3 | *copB* | Copper resistance protein B precursor | *Weissella thailandensis* |
|  | YP_004929442.1 | 3 | *copB* | Copper resistance protein B precursor | *Pseudoxanthomonas spadix* |
|  | ZP_04661420.1 | 3 | *copC* | Copper resistance protein | *Acinetobacter baumannii* |
|  | YP_004929429.1 | 3 | *copC* | Copper resistance protein | *Pseudoxanthomonas spadix* |
|  | ZP_03541661.1 | 3 | *copD* | Copper resistance protein | *Comamonas testosteroni* |
|  | YP_003749519.1 | 3 | *cusR* | Copper resistance transcriptional regulator | *Ralstonia solanacearum* |
|  | YP_829197.1 | 3 | Others | Copper resistance D domain-containing protein | *Arthrobacter* sp. |
|  | ZP_04762051.1 | 3 | Others | Copper resistance outer-membrane lipoprotein | *Acidovorax delafieldii* |
|  | ZP_05136509.1 | 3 | Others | response regulator in two-component regulatory system with CusS, regulation of copper resistance | *Stenotrophomonas* sp. |
|  | YP_001195414.1 | 2 | *copA* | Multicopper oxidase type 3 | *Flavobacterium johnsoniae* |
|  | YP_001242334.1 | 2 | *copA* | Multicopper oxidase type 3 | *Bradyrhizobium* sp. |
|  | YP_002291272.1 | 2 | *copA* | Multicopper oxidase type 3 | *Ornithobacterium rhinotracheale* |
|  | ZP_03543174.1 | 2 | *copA* | Multicopper oxidase type 3 | *Comamonas testosteroni* |
|  | ZP_06067630.1 | 2 | *copA* | Multicopper oxidase type 3 | *Acinetobacter junii* |
|  | ZP_06070830.1 | 2 | *copA* | Multicopper oxidase type 3 | *Acinetobacter lwoffii* |
|  | ZP_07087208.1 | 2 | *copA* | Multicopper oxidase type 3 | *Chryseobacterium gleum* |
|  | ZP_07089077.1 | 2 | *copA* | Multicopper oxidase type 3 | *Chryseobacterium gleum* |
|  | YP_004273982.1 | 2 | *copA* | Multicopper oxidase type 3 | *Pedobacter saltans* |
|  | AEG71068.1 | 2 | *copA* | Multicopper oxidase type 3 | *Ralstonia solanacearum* |
|  | YP_497409.1 | 2 | *copA* | Multicopper oxidase type 3 | *Novosphingobium aromaticivorans* |
|  | YP_617497.1 | 2 | *copB* | Copper resistance protein B precursor | *Sphingopyxis alaskensis* |
|  | YP_001714980.1 | 2 | *copB* | Copper resistance protein B precursor | *Acinetobacter baumannii* |
|  | YP_002128720.1 | 2 | *copB* | Copper resistance protein B precursor | *Phenylobacterium zucineum* |
|  | ZP_08389863.1 | 2 | *copB* | Copper resistance protein B precursor | *Sphingomonas* sp. |
|  | ZP_08390471.1 | 2 | *copB* | Copper resistance protein B precursor | *Sphingomonas* sp*.* |
|  | ZP_01303665.1 | 2 | *copB* | Copper resistance protein B precursor | *Sphingomonas* sp. |
|  | ZP_03541662.1 | 2 | *copC* | Copper resistance protein | *Comamonas testosteroni* |
|  | ZP_08071275.1 | 2 | *copC* | Copper resistance protein | *Methylocystis* sp. |
|  | ZP_06070844.1 | 2 | *copD* | Putative copper export protein | *Acinetobacter lwoffii* |
|  | ZP_07677917.1 | 2 | *copD* | Putative copper export protein | *Ralstonia* sp. |
|  | YP_002890929.1 | 2 | *copK* | Copper resistance protein | *Thauera* sp. |
|  | YP_145674.1 | 2 | *copL* | Putative type II restriction enzyme | *Cupriavidus metallidurans* |
|  | AEG71069.1 | 2 | *copR* | Regulatory system with cops, regulation of copper resistance | *Ralstonia solanacearum* |
|  | YP_002961462.1 | 2 | *cusR* | Copper resistance transcriptional regulator | *Methylobacterium extorquens* |
|  | ACB12991.1 | 2 | Others | Outer membrane efflux protein | *Aquabacterium* sp. |
|  | ZP_01961984.1 | 2 | Others | Uncharacterized protein involved in copper resistance | *Bacteroides caccae* |
|  | YP_002128735.1 | 1 | *copA* | Copper resistance protein | *Phenylobacterium zucineum* |
|  | YP_615837.1 | 1 | *copA* | Copper-resistance protein | *Sphingopyxis alaskensis* |
|  | YP_617496.1 | 1 | *copA* | Copper-resistance protein | *Sphingopyxis alaskensis* |
|  | ABO10259.1 | 1 | *copA* | Copper-resistance protein | Uncultured bacterium |
|  | ABO10316.1 | 1 | *copA* | Copper-resistance protein | Uncultured bacterium |
|  | ABO10328.1 | 1 | *copA* | Copper-resistance protein | Uncultured bacterium |
|  | ABO10334.1 | 1 | *copA* | Copper-resistance protein | Uncultured bacterium |
|  | ABO10234.2 | 1 | *copA* | Copper-resistance protein | Uncultured bacterium |
|  | ABO10338.2 | 1 | *copA* | Copper-resistance protein | Uncultured bacterium |
|  | YP_001683977.1 | 1 | *copA* | Copper-resistance protein | *Caulobacter* sp. |
|  | YP_617463.1 | 1 | *copA* | Multicopper oxidase type 3 | *Sphingopyxis alaskensis* |
|  | YP_001220465.1 | 1 | *copA* | Multicopper oxidase type 3 | *Bradyrhizobium* sp. |
|  | YP_001440265.1 | 1 | *copA* | Multicopper oxidase type 3 | *Cronobacter sakazakii* |
|  | YP_001580520.1 | 1 | *copA* | Multicopper oxidase type 3 | *Burkholderia multivorans* |
|  | YP_002754313.1 | 1 | *copA* | Multicopper oxidase type 3 | *Acidobacterium capsulatum* |
|  | ZP_05133614.1 | 1 | *copA* | Multicopper oxidase type 3 | *Stenotrophomonassp.* |
|  | YP_003067026.1 | 1 | *copA* | Multicopper oxidase type 3 | *Methylobacterium extorquens* |
|  | ZP_07081131.1 | 1 | *copA* | Multicopper oxidase type 3 | *Sphingobacterium spiritivorum* |
|  | ZP_08274379.1 | 1 | *copA* | Multicopper oxidase type 3 | *Oxalobacteraceae bacterium* |
|  | ZP_08389867.1 | 1 | *copA* | Multicopper oxidase type 3 | *Sphingomonas* sp. |
|  | YP_004535037.1 | 1 | *copA* | Multicopper oxidase type 3 | *Novosphingobium* sp. |
|  | ZP_08952861.1 | 1 | *copA* | Multicopper oxidase type 3 | *Rhodanobacter* sp. |
|  | YP_095068.1 | 1 | *copA* | Multicopper oxidase type 3 | *Legionella pneumophila* |
|  | ZP_01304008.1 | 1 | *copA* | Multicopper oxidase type 3 | *Sphingomonas* sp. |
|  | ZP_03821657.1 | 1 | *copB* | Copper resistance protein B precursor | *Acinetobacter* sp. |
|  | ZP_05136243.1 | 1 | *copB* | Copper resistance protein B precursor | *Stenotrophomonas* sp. |
|  | ZP_06066683.1 | 1 | *copB* | Copper resistance protein B precursor | *Acinetobacter junii* |
|  | ZP_06067631.1 | 1 | *copB* | Copper resistance protein B precursor | *Acinetobacter junii* |
|  | YP_003391634.1 | 1 | *copB* | Copper resistance protein B precursor | *Spirosoma linguale* |
|  | ZP_06726396.1 | 1 | *copB* | Copper resistance protein B precursor | *Acinetobacter haemolyticus* |
|  | ZP_08268034.1 | 1 | *copB* | Copper resistance protein B precursor | *Brevundimonas diminuta* |
|  | ZP_08389827.1 | 1 | *copB* | Copper resistance protein B precursor | *Sphingomonas* sp. |
|  | YP_004416263.1 | 1 | *copB* | Copper resistance protein B precursor | *Pusillimonas* sp. |
|  | ZP_08437415.1 | 1 | *copB* | Copper resistance protein B precursor | *Acinetobacter baumannii* |
|  | ZP_01303653.1 | 1 | *copB* | Copper resistance protein B precursor | *Sphingomonas* sp. |
|  | YP_001561476.1 | 1 | *copC* | Copper resistance protein | *Delftia acidovorans* |
|  | YP_001714986.1 | 1 | *copC* | Copper resistance protein | *Acinetobacter baumannii* |
|  | ZP_03821650.1 | 1 | *copC* | Copper resistance protein | *Acinetobacter* sp. |
|  | YP_114592.1 | 1 | *copC* | Copper resistance protein | *Methylococcus capsulatus* |
|  | ZP_06067620.1 | 1 | *copC* | Copper resistance protein | *Acinetobacter junii* |
|  | YP_003020097.1 | 1 | *copD* | Copper resistance protein | *Geobacter* sp. |
|  | ZP_07047686.1 | 1 | *copD* | Copper resistance protein | *Comamonas testosteroni* |
|  | ZP_07047701.1 | 1 | *copD* | Copper resistance protein | *Comamonas testosteroni* |
|  | YP_004514344.1 | 1 | *copD* | Copper resistance protein | *Methylomonas methanica* |
|  | XP_003343619.1 | 1 | *copD* | Copper resistance protein | *Sordaria macrospora* |
|  | YP_001561475.1 | 1 | *copD* | Putative copper export protein | *Delftia acidovorans* |
|  | ZP_04661421.1 | 1 | *copD* | Putative copper export protein | *Acinetobacter baumannii* |
|  | AEC46571.1 | 1 | *copF* | Copper resistance protein F | *Xanthomonas alfalfae* |
|  | CBJ40258.1 | 1 | *copR* | Copper resistance transcriptional | *Ralstonia solanacearum* |
|  | YP_001239427.1 | 1 | *cusB* | Cation efflux system protein | *Bradyrhizobium* sp. |
|  | NP_752589.1 | 1 | *cusC* | Copper/silver efflux system outer membrane protein | *Escherichia coli* |
|  | NP_836193.1 | 1 | *cusC* | Copper/silver efflux system outer membrane protein | *Shigella flexneri* |
|  | YP_001338357.1 | 1 | *cusR* | Copper resistance transcriptional regulator | *Klebsiella pneumoniae* |
|  | YP_003544163.1 | 1 | *cusR* | Copper resistance transcriptional regulator | *Sphingobium japonicum* |
|  | YP_003612460.1 | 1 | *cusR* | Copper resistance transcriptional regulator | *Enterobacter cloacae* |
|  | ZP_08411666.1 | 1 | *cusR* | Copper resistance transcriptional regulator | *Pseudoalteromonas haloplanktis* |
|  | ZP_03028866.1 | 1 | *cutF* | Copper homeostasis protein | *Escherichia coli* |
|  | YP_309232.1 | 1 | *cutF* | Copper homeostasis and adhesion lipoprotein | *Shigella sonnei* |
|  | YP_001336013.1 | 1 | *yebZ* | Putative copper export protein | *Klebsiella pneumoniae* subsp. |
|  | ZP_06551812.1 | 1 | Others | Copper homeostasis protein | *Klebsiella* sp. |
|  | YP_004655142.1 | 1 | Others | Copper homeostasis protein | *Runella slithyformis* |
|  | YP_001899346.1 | 1 | Others | Copper resistance D domain-containing protein | *Ralstonia pickettii* |
|  | YP_986942.1 | 1 | Others | Copper resistance outer-membrane lipoprotein | *Acidovorax* sp. |
|  | YP_002553639.1 | 1 | Others | Copper resistance outer-membrane lipoprotein | *Acidovorax ebreus* |
|  | YP_001899289.1 | 1 | Others | Copper resistance protein | *Ralstonia pickettii* |
|  | ZP_07677916.1 | 1 | Others | Copper resistance protein | *Ralstonia* sp. |
|  | YP_004592546.1 | 1 | Others | Lipoprotein involved with copper homeostasis and adhesion | *Enterobacter aerogenes* |
|  | CBL24844.1 | 1 | Others | Uncharacterized protein involved in copper resistance | *Ruminococcus torques* |
| S7 | YP_003496669.1 | 17 | *ACR3* | Arsenic membrane transporter | *Deferribacter desulfuricans* |
|  | YP_003693513.1 | 12 | *ACR3* | Arsenic membrane transporter | *Starkeya novella* |
|  | YP_002136352.1 | 10 | *ACR3* | Arsenic membrane transporter | *Anaeromyxobacter* sp. |
|  | YP_003021684.1 | 10 | *ACR3* | Arsenic membrane transporter | *Geobacter* sp. |
|  | AAY85171.1 | 9 | *arsB* | Arsenite transmembrane pump | *Leptospirillum ferriphilum* |
|  | NP_902110.1 | 8 | *arsB* | Arsenic membrane transporter | *Chromobacterium violaceum* |
|  | YP_111445.1 | 7 | *ACR3* | Arsenic membrane transporter | *Burkholderia pseudomallei* |
|  | YP_001301411.1 | 5 | *ACR3* | Arsenic membrane transporter | *Bacteroides vulgatus* |
|  | YP_001892366.1 | 4 | *ACR3* | Arsenic membrane transporter | *Ralstonia pickettii* |
|  | YP_002355501.1 | 4 | *ACR3* | Arsenic membrane transporter | *Thauera* sp. |
|  | YP_004013809.1 | 4 | *ACR3* | Arsenic membrane transporter | *Rhodomicrobium vannielii* |
|  | YP_001951397.1 | 3 | *ACR3* | Arsenic membrane transporter | *Geobacter lovleyi* |
|  | YP_002457706.1 | 3 | *ACR3* | Arsenic membrane transporter | *Desulfitobacterium hafniense* |
|  | YP_002892220.1 | 3 | *ACR3* | Arsenic membrane transporter | *Tolumonas auensis* |
|  | YP_004077269.1 | 3 | *ACR3* | Arsenic membrane transporter | *Mycobacterium gilvum* |
|  | ADZ05767.1 | 3 | *ACR3* | Arsenic membrane transporter | *Acinetobacter baumannii* |
|  | ZP_08235443.1 | 3 | *ACR3* | Arsenic membrane transporter | *Streptomyces griseus* |
|  | YP_462982.1 | 3 | *ACR3* | Arsenic membrane transporter | *Syntrophus aciditrophicus* |
|  | ABF22610.1 | 3 | *arsA* | Anion-transporting ATPase | *Ochrobactrum tritici* |
|  | XP_001182180.1 | 3 | *arsH* | Arsenic resistance protein | *Strongylocentrotus purpuratus* |
|  | ZP_03824810.1 | 3 | *arsH* | Arsenic resistance protein | *Acinetobacter* sp. |
|  | NP_569191.1 | 2 | *ACR3* | Arsenic membrane transporter | *Listeria innocua* |
|  | YP_002422897.1 | 2 | *ACR3* | Arsenic membrane transporter | *Methylobacterium chloromethanicum* |
|  | YP_002507030.1 | 2 | *ACR3* | Arsenic membrane transporter | *Clostridium cellulolyticum* |
|  | ZP_03592353.1 | 2 | *ACR3* | Arsenic membrane transporter | *Bacillus subtilis* subsp. |
|  | YP_003756459.1 | 2 | *ACR3* | Arsenic membrane transporter | *Hyphomicrobium denitrificans* |
|  | YP_004319955.1 | 2 | *ACR3* | Arsenic membrane transporter | *Sphingobacterium* sp. |
|  | ABF22612.1 | 2 | *arsB* | Arsenite membrane pump | *Ochrobactrum tritici* |
|  | AAY85167.1 | 2 | *arsC* | Arsenate reductase-like protein | *Leptospirillum ferriphilum* |
|  | YP_703329.1 | 1 | *ACR3* | Arsenic membrane transporter | *Rhodococcus jostii* |
|  | ZP_01739410.1 | 1 | *ACR3* | Arsenic membrane transporter | *Marinobacter* sp. |
|  | YP_001869691.1 | 1 | *ACR3* | Arsenic membrane transporter | *Nostoc punctiforme* |
|  | YP_001972211.1 | 1 | *ACR3* | Arsenic membrane transporter | *Stenotrophomonas maltophilia* |
|  | YP_002419884.1 | 1 | *ACR3* | Arsenic membrane transporter | *Methylobacterium chloromethanicum* |
|  | YP_002988705.1 | 1 | *ACR3* | Arsenic membrane transporter | *Dickeya dadantii* |
|  | ZP_05649774.1 | 1 | *ACR3* | Arsenic membrane transporter | *Enterococcus gallinarum* |
|  | YP_003340106.1 | 1 | *ACR3* | Arsenic membrane transporter | *Streptosporangium roseum* |
|  | YP_003989268.1 | 1 | *ACR3* | Arsenic membrane transporter | *Geobacillus* sp. |
|  | YP_004099318.1 | 1 | *ACR3* | Arsenic membrane transporter | *Intrasporangium calvum* |
|  | NP_856322.1 | 1 | *ACR3* | Arsenic membrane transporter | *Mycobacterium bovis* |
|  | YP_004264406.1 | 1 | *ACR3* | Arsenic membrane transporter | *Syntrophobotulus glycolicus* |
|  | ZP_08288615.1 | 1 | *ACR3* | Arsenic membrane transporter | *Streptomyces griseoaurantiacus* |
|  | ZP_00997259.1 | 1 | *ACR3* | Arsenic membrane transporter | *Janibacter* sp. |
|  | ABF48394.1 | 1 | *ACR3* | Arsenic membrane transporter | *Ochrobactrum tritici* |
|  | AAU03124.1 | 1 | *arsC* | Arsenate reductase | *Pseudomonas stutzeri* |
|  | XP_001189774.1 | 1 | *arsH* | Arsenic resistance protein | *Strongylocentrotus purpuratus* |
|  | ZP_07044498.1 | 1 | *arsH* | Arsenic resistance protein | *Comamonas testosteroni* |
|  | YP_003977145.1 | 1 | *arsH* | Arsenic resistance protein | *Achromobacter xylosoxidans* |
|  | YP_004792856.1 | 1 | *arsH* | Arsenic resistance protein | *Stenotrophomonas maltophilia* |
|  | AAY85170.1 | 1 | Others | CBS domain-like protein | *Leptospirillum ferriphilum* |
|  | ZP_07673472.1 | 20 | *czcA* | Cobalt-zinc-cadmium resistance protein | *Ralstonia* sp. |
|  | ZP_03543140.1 | 16 | *czcA* | Cobalt-zinc-cadmium resistance protein | *Comamonas testosteroni* |
|  | YP_004042151.1 | 13 | *acrB/D/F* | Heavy metal efflux pump | *Paludibacter propionicigenes* |
|  | YP_001099897.1 | 13 | *czcA* | Cobalt-zinc-cadmium resistance protein | *Herminiimonas arsenicoxydans* |
|  | ZP_08275386.1 | 9 | *czcA* | Cobalt-zinc-cadmium resistance protein | *Oxalobacteraceae bacterium* |
|  | NCCA_ALCXX | 9 | *nccA* | Nickel-cobalt-cadmium resistance protein nccA | *Achromobacter xylosoxidans* |
|  | ZP_06061604.1 | 9 | Others | Cobalt-zinc-cadmium efflux pump | *Acinetobacter johnsonii* |
|  | ZP_08506022.1 | 8 | *czcA* | Cobalt-zinc-cadmium resistance protein | *Methyloversatilis universalis* |
|  | ZP_08072272.1 | 7 | *czcA* | Cobalt-zinc-cadmium resistance protein | *Methylocystis* sp. |
|  | YP_145594.1 | 6 | *czcB* | Cobalt-zinc-cadmium resistance protein | *Cupriavidus metallidurans* |
|  | NP_421193.1 | 5 | *acrB/D/F* | Heavy metal efflux pump | *Caulobacter crescentus* |
|  | YP_003777808.1 | 5 | *czcA* | Cobalt-zinc-cadmium resistance protein | *Herbaspirillum seropedicae* |
|  | ZP_08441696.1 | 5 | *czcA* | Cobalt-zinc-cadmium resistance protein | *Acinetobacter baumannii* |
|  | YP_145595.1 | 5 | *czcA* | Cobalt-zinc-cadmium resistance protein | *Cupriavidus metallidurans* |
|  | AF333961_2 | 4 | *cadA* | Cadmium resistance protein | *Pseudomonas putida* |
|  | YP_931731.1 | 4 | *czcA* | Cobalt-zinc-cadmium resistance protein | *Azoarcus* sp. |
|  | YP_003280424.1 | 4 | *czcA* | Cobalt-zinc-cadmium resistance protein | *Comamonas testosteroni* |
|  | YP_001796416.1 | 3 | *czcA* | Cobalt-zinc-cadmium resistance protein | *Cupriavidus taiwanensis* |
|  | YP_001974238.1 | 3 | *czcA* | Cobalt-zinc-cadmium resistance protein | *Stenotrophomonas maltophilia* |
|  | ZP_03821708.1 | 3 | *czcA* | Cobalt-zinc-cadmium resistance protein | *Acinetobacter* sp. |
|  | YP_003122408.1 | 3 | *czcA* | Cobalt-zinc-cadmium resistance protein | *Chitinophaga pinensis* |
|  | ZP_08272425.1 | 3 | *czcA* | Cobalt-zinc-cadmium resistance protein | *Gamma proteobacterium* |
|  | ZP_08276460.1 | 3 | *czcA* | Cobalt-zinc-cadmium resistance protein | *Oxalobacteraceae bacterium* |
|  | YP_262295.1 | 3 | *czcA* | Cobalt-zinc-cadmium resistance protein | *Pseudomonas fluorescens* |
|  | YP_004451316.1 | 2 | *acrB/D/F* | Heavy metal efflux pump | *Haliscomenobacter hydrossis* |
|  | YP_954439.1 | 2 | *cadD* | Cadmium resistance transporter | *Mycobacterium vanbaalenii* |
|  | YP_002560802.1 | 2 | *cadD* | Cadmium binding protein | *Macrococcus caseolyticus* |
|  | ZP_07091299.1 | 2 | *cadD* | Cadmium resistance transporter | *Corynebacterium genitalium* |
|  | ZP_08250611.1 | 2 | *cadD* | Cadmium resistance family protein | *Dialister micraerophilus* |
|  | YP_003602529.1 | 2 | *cusC* | Efflux system outer membrane protein | *Enterobacter cloacae* |
|  | ZP_02156302.1 | 2 | *czcA* | Cobalt-zinc-cadmium resistance protein | *Shewanella benthica* |
|  | ZP_06729092.1 | 2 | *czcA* | Cobalt-zinc-cadmium resistance protein | *Acinetobacter haemolyticus* |
|  | EFV84518.1 | 2 | *czcA* | Cobalt-zinc-cadmium resistance protein | *Achromobacter xylosoxidans* |
|  | EGH38844.1 | 2 | *czcA* | Cobalt-zinc-cadmium resistance protein | *Escherichia coli* |
|  | EHC73733.1 | 2 | *czcA* | Cobalt-zinc-cadmium resistance protein | *Salmonella enterica* |
|  | YP_002233336.1 | 2 | *czcA* | Cobalt-zinc-cadmium resistance transporter protein | *Burkholderia cenocepacia* |
|  | YP_004046468.1 | 2 | *MMT1* | Cation efflux protein | *Riemerella anatipestifer* |
|  | YP_002870366.1 | 1 | *acrB/D/F* | Heavy metal efflux pump | *Pseudomonas fluorescens* |
|  | YP_931772.1 | 1 | *cadA* | Cadmium resistance protein | *Pseudomonas putida* |
|  | ABR09922.1 | 1 | *cadA* | Cadmium resistance ATPase | *Bacillus* sp. |
|  | YP_263296.1 | 1 | *cadA* | Cadmium resistance protein | *Lactococcus lactis* |
|  | YP_040152.1 | 1 | *cadC* | Cadmium efflux system accessory protein | *Staphylococcus aureus* subsp. |
|  | YP_954047.1 | 1 | *cadD* | Cadmium resistance transporter | *Mycobacterium vanbaalenii* |
|  | YP_001134445.1 | 1 | *cadD* | Cadmium resistance transporter | *Mycobacterium gilvum* |
|  | YP_001134476.1 | 1 | *cadD* | Cadmium resistance transporter | *Mycobacterium gilvum* |
|  | ZP_03305283.1 | 1 | *cadD* | Cadmium resistance transporter | *Anaerococcus hydrogenalis* |
|  | ZP_06501487.1 | 1 | *cadD* | Cadmium resistance transporter | *Micrococcus luteus* |
|  | YP_003635466.1 | 1 | *cadD* | Cadmium resistance transporter | *Cellulomonas flavigena* |
|  | ZP_07664505.1 | 1 | *cadD* | Cadmium resistance transporter | *Atopobium vaginae* |
|  | ZP_07698549.1 | 1 | *cadD* | Cadmium resistance transporter | *Lactobacillus iners* |
|  | NP_744558.1 | 1 | *czcA* | Cobalt-zinc-cadmium resistance protein | *Pseudomonas putida* |
|  | ZP_07007253.1 | 1 | *czcA* | Cobalt-zinc-cadmium resistance protein | *Pseudomonas savastanoi* |
|  | YP_004258364.1 | 1 | *czcA* | Cobalt-zinc-cadmium resistance protein | *Bacteroides salanitronis* |
|  | AEA07975.1 | 1 | *czcA* | Cadmium resistance protein | *Proteus vulgaris* |
|  | YP_004738505.1 | 1 | *czcA* | Cobalt-zinc-cadmium resistance protein | *Zobellia galactanivorans* |
|  | EHC89288.1 | 1 | *czcA* | Cobalt-zinc-cadmium resistance protein | *Salmonella enterica* |
|  | ZP_08262059.1 | 1 | *nccA* | Nickel-cobalt-cadmium resistance protein nccA | *Asticcacaulis biprosthecum* |
|  | YP_004653321.1 | 1 | *nccA* | Nickel-cobalt-cadmium resistance protein nccA | *Parachlamydia acanthamoebae* |
|  | NP_763630.1 | 1 | Others | Cadmium resistance protein B | *Staphylococcus epidermidis* |
|  | NP_268159.1 | 1 | Others | Metal transporting ATPase | *Lactococcus lactis* subsp. |
|  | YP_004096061.1 | 1 | Others | ATPase P | *Bacillus cellulosilyticus* |
|  | YP_004753177.1 | 1 | Others | Heavy metal response regulator | *Collimonas fungivorans* |
|  | YP_109973.1 | 1 | Others | MerR family regulatory protein | *Burkholderia pseudomallei* |
|  | AAF22880.1 | 1 | Others | Putative inner membrane protein | *Bradyrhizobium japonicum* |
|  | ZP_08506054.1 | 35 | *copA* | Multicopper oxidase type 3 | *Methyloversatilis universalis* |
|  | YP_004929441.1 | 16 | *copA* | Multicopper oxidase type 3 | *Pseudoxanthomonas spadix* |
|  | ZP_08274379.1 | 15 | *copA* | Multicopper oxidase type 3 | *Oxalobacteraceae bacterium* |
|  | ZP_05361756.1 | 14 | *copA* | Multicopper oxidase type 3 | *Acinetobacter radioresistens* |
|  | CAC07984.1 | 13 | *copF* | CopF Cu-ATPase | *Ralstonia metallidurans* |
|  | ZP_07567309.1 | 10 | *copB* | Copper resistance protein B precursor | *Enterococcus faecalis* |
|  | ZP_06073977.1 | 10 | *copD* | Copper resistance protein | *Acinetobacter radioresistens* |
|  | ZP_07677914.1 | 8 | *copA* | Multicopper oxidase type 3 | *Ralstonia* sp. |
|  | ZP_01303652.1 | 8 | *copA* | Multicopper oxidase type 3 | *Sphingomonas* sp. |
|  | ZP_08506053.1 | 8 | *copB* | Copper resistance protein B precursor | *Methyloversatilis universalis* |
|  | YP_004929429.1 | 7 | *copC* | Copper resistance protein | *Pseudoxanthomonas spadix* |
|  | ZP_08411666.1 | 7 | *cusR* | Copper resistance transcriptional regulator | *Pseudoalteromonas haloplanktis* |
|  | YP_004346534.1 | 6 | *copA* | Multicopper oxidase type 3 | *Fluviicola taffensis* |
|  | YP_124656.1 | 6 | *copA* | Multicopper oxidase type 3 | *Legionella pneumophila* |
|  | ZP_08390471.1 | 6 | *copB* | Copper resistance protein B precursor | *Sphingomonas* sp. |
|  | YP_004929442.1 | 6 | *copB* | Copper resistance protein B precursor | *Pseudoxanthomonas spadix* |
|  | YP_746278.1 | 5 | *copA* | Multicopper oxidase type 3 | *Nitrosomonas eutropha* |
|  | ZP_06686120.1 | 5 | *copA* | Multicopper oxidase type 3 | *Achromobacter piechaudii* |
|  | ZP_08389867.1 | 5 | *copA* | Multicopper oxidase type 3 | *Sphingomonas* sp. |
|  | ZP_05361757.1 | 5 | *copB* | Copper resistance protein B precursor | *Acinetobacter radioresistens* |
|  | EGO37246.1 | 5 | *copD* | Copper resistance protein | *Mycobacterium avium* |
|  | ZP_03543174.1 | 4 | *copA* | Multicopper oxidase type 3 | *Comamonas testosteroni* |
|  | ZP_08433026.1 | 4 | *copA* | Multicopper oxidase type 3 | *Acinetobacter baumannii* |
|  | ZP_07043165.1 | 4 | *copB* | Copper resistance protein B precursor | *Comamonas testosteroni* |
|  | ZP_08389863.1 | 4 | *copB* | Copper resistance protein B precursor | *Sphingomonas* sp. |
|  | AEC46571.1 | 4 | *copF* | Copper resistance protein F | *Xanthomonas alfalfae* |
|  | ZP_04762051.1 | 4 | Others | Copper resistance outer-membrane lipoprotein | *Acidovorax delafieldii* |
|  | YP_001220465.1 | 3 | *copA* | Multicopper oxidase type 3 | *Bradyrhizobium* sp. |
|  | YP_002128735.1 | 3 | *copA* | Multicopper oxidase type 3 | *Phenylobacterium zucineum* |
|  | ZP_08406915.1 | 3 | *copA* | Multicopper oxidase type 3 | *Hylemonella gracilis* |
|  | ZP_08952861.1 | 3 | *copA* | Multicopper oxidase type 3 | *Rhodanobacter* sp. |
|  | ZP_05136243.1 | 3 | *copB* | Copper resistance protein B precursor | *Stenotrophomonas* sp. |
|  | ZP_06064053.1 | 3 | *copB* | Copper resistance protein B precursor | *Acinetobacter johnsonii* |
|  | ZP_08273252.1 | 3 | *copB* | Copper resistance protein B precursor | *Oxalobacteraceae bacterium* |
|  | YP_001561475.1 | 3 | *copD* | Putative copper export protein | *Delftia acidovorans* |
|  | ZP_08390480.1 | 3 | *copD* | Copper resistance protein | *Sphingomonas* sp. |
|  | XP_003343619.1 | 3 | *copD* | Copper resistance protein | *Sordaria macrospora* |
|  | CCE54380.1 | 3 | *copD* | Copper resistance protein | *Corynebacterium casei* |
|  | YP_004929428.1 | 3 | *copD* | Copper resistance protein | *Pseudoxanthomonas spadix* |
|  | YP_001098874.1 | 3 | *cusR* | Copper resistance transcriptional regulator | *Herminiimonas arsenicoxydans* |
|  | YP_001561483.1 | 3 | Others | Heavy metal sensor signal transduction histidine kinase | *Delftia acidovorans* |
|  | ZP_05111242.1 | 3 | Others | Copper efflux ATPase | *Legionella drancourtii* |
|  | YP_617463.1 | 2 | *copA* | Multicopper oxidase type 3 | *Sphingopyxis alaskensis* |
|  | YP_985537.1 | 2 | *copA* | Multicopper oxidase type 3 | *Acidovorax sp.* |
|  | ZP_01886263.1 | 2 | *copA* | Multicopper oxidase type 3 | *Pedobacter sp.* |
|  | YP_001419480.1 | 2 | *copA* | Multicopper oxidase type 3 | *Xanthobacter autotrophicus* |
|  | YP_001899418.1 | 2 | *copA* | Multicopper oxidase type 3 | *Ralstonia pickettii* |
|  | YP_001990114.1 | 2 | *copA* | Multicopper oxidase type 3 | *Rhodopseudomonas palustris* |
|  | ZP_06066684.1 | 2 | *copA* | Multicopper oxidase type 3 | *Acinetobacter junii* |
|  | ZP_06067630.1 | 2 | *copA* | Multicopper oxidase type 3 | *Acinetobacter junii* |
|  | ZP_07082318.1 | 2 | *copA* | Multicopper oxidase type 3 | *Sphingobacterium spiritivorum* |
|  | YP_004046465.1 | 2 | *copA* | Multicopper oxidase type 3 | *Riemerella anatipestifer* |
|  | YP_004682352.1 | 2 | *copA* | Multicopper oxidase type 3 | *Cupriavidus necator* |
|  | ZP_01303666.1 | 2 | *copA* | Multicopper oxidase type 3 | *Sphingomonas* sp. |
|  | ZP_02185059.1 | 2 | *copB* | Copper resistance protein B precursor | *Carnobacterium* sp. |
|  | ZP_06067631.1 | 2 | *copB* | Copper resistance protein B precursor | *Acinetobacter junii* |
|  | ZP_08180218.1 | 2 | *copB* | Copper resistance protein B precursor | *Xanthomonas vesicatoria* |
|  | ZP_05111649.1 | 2 | *copB* | Copper resistance protein B | *Legionella drancourtii* |
|  | ZP_06067620.1 | 2 | *copC* | Copper resistance protein | *Acinetobacter junii* |
|  | ZP_08634378.1 | 2 | *copC* | Copper resistance protein | *Acidiphilium* sp. |
|  | YP_829197.1 | 2 | *copD* | Copper resistance protein | *Arthrobacter* sp. |
|  | ZP_08389832.1 | 2 | *copD* | Copper resistance protein | *Sphingomonas* sp. |
|  | YP_004514344.1 | 2 | *copD* | Copper resistance protein | *Methylomonas methanica* |
|  | YP_497414.1 | 2 | *copD* | Copper resistance protein | *Novosphingobium aromaticivorans* |
|  | AEG71069.1 | 2 | *copR* | Copper resistance transcriptional regulator | *Ralstonia solanacearum* |
|  | YP_004391983.1 | 2 | *cusF* | Copper/silver resistance periplasmic protein | *Aeromonas veronii* |
|  | YP_003749519.1 | 2 | *cusR* | Copper resistance transcriptional regulator | *Ralstonia solanacearum* |
|  | YP_004676151.1 | 2 | *cusR* | Copper resistance transcriptional regulator | *Hyphomicrobium* sp. |
|  | YP_001481465.1 | 2 | *silC* | Copper/silver efflux system outer membrane protein | *Escherichia coli* |
|  | YP_001220520.1 | 2 | Others | putative copper resistance protein A precursor | *Bradyrhizobium* sp. |
|  | YP_615837.1 | 1 | *copA* | Multicopper oxidase type 3 | *Sphingopyxis alaskensis* |
|  | YP_760413.1 | 1 | *copA* | Multicopper oxidase type 3 | *Hyphomonas neptunium* |
|  | ABO10209.1 | 1 | *copA* | Multicopper oxidase type 3 | *uncultured bacterium* |
|  | ABO10326.1 | 1 | *copA* | Multicopper oxidase type 3 | *uncultured bacterium* |
|  | ABO10328.1 | 1 | *copA* | Multicopper oxidase type 3 | *uncultured bacterium* |
|  | YP_001192548.1 | 1 | *copA* | Multicopper oxidase type 3 | *Flavobacterium johnsoniae* |
|  | YP_001195414.1 | 1 | *copA* | Multicopper oxidase type 3 | *Flavobacterium johnsoniae* |
|  | YP_001242334.1 | 1 | *copA* | Multicopper oxidase type 3 | *Bradyrhizobium* sp. |
|  | ZP_01898887.1 | 1 | *copA* | Multicopper oxidase type 3 | *Moritella* sp. |
|  | ABO10337.2 | 1 | *copA* | Multicopper oxidase type 3 | *uncultured bacterium* |
|  | ABO10338.2 | 1 | *copA* | Multicopper oxidase type 3 | *uncultured bacterium* |
|  | YP_001381429.1 | 1 | *copA* | Multicopper oxidase type 3 | *Anaeromyxobacter* sp. |
|  | YP_001411626.1 | 1 | *copA* | Multicopper oxidase type 3 | *Parvibaculum lavamentivorans* |
|  | YP_001580520.1 | 1 | *copA* | Multicopper oxidase type 3 | *Burkholderia multivorans* |
|  | YP_001696272.1 | 1 | *copA* | Multicopper oxidase type 3 | *Lysinibacillus sphaericus* |
|  | YP_001899289.1 | 1 | *copA* | Multicopper oxidase type 3 | *Ralstonia pickettii* |
|  | YP_002130318.1 | 1 | *copA* | Multicopper oxidase type 3 | *Phenylobacterium zucineum* |
|  | YP_002291272.1 | 1 | *copA* | Multicopper oxidase type 3 | *Ornithobacterium rhinotracheale* |
|  | YP_002754313.1 | 1 | *copA* | Multicopper oxidase type 3 | *Acidobacterium capsulatum* |
|  | ZP_04101656.1 | 1 | *copA* | Multicopper oxidase type 3 | *Bacillus thuringiensis serovar berliner* |
|  | ZP_04661415.1 | 1 | *copA* | Multicopper oxidase type 3 | *Acinetobacter baumannii* |
|  | YP_002962972.1 | 1 | *copA* | Multicopper oxidase type 3 | *Methylobacterium extorquens* |
|  | YP_002973578.1 | 1 | *copA* | Multicopper oxidase type 3 | *Ralstonia pickettii* |
|  | ZP_05823239.1 | 1 | *copA* | Multicopper oxidase type 3 | *Acinetobacter* sp. |
|  | CAZ89693.1 | 1 | *copA* | Multicopper oxidase type 3 | *Thiomonas* sp. |
|  | YP_003594378.1 | 1 | *copA* | Multicopper oxidase type 3 | *Caulobacter segnis* |
|  | YP_003618340.1 | 1 | *copA* | Multicopper oxidase type 3 | *Legionella pneumophila* |
|  | ZP_07081131.1 | 1 | *copA* | Multicopper oxidase type 3 | *Sphingobacterium spiritivorum* |
|  | ZP_07089169.1 | 1 | *copA* | Multicopper oxidase type 3 | *Chryseobacterium gleum* |
|  | YP_003977182.1 | 1 | *copA* | Multicopper oxidase type 3 | *Achromobacter xylosoxidans* |
|  | YP_004273982.1 | 1 | *copA* | Multicopper oxidase type 3 | *Pedobacter saltans* |
|  | AEG70972.1 | 1 | *copA* | Multicopper oxidase type 3 | *Ralstonia solanacearum* |
|  | YP_004843209.1 | 1 | *copA* | Multicopper oxidase type 3 | *Flavobacterium branchiophilum* |
|  | YP_095068.1 | 1 | *copA* | Multicopper oxidase type 3 | *Legionella pneumophila* |
|  | YP_497409.1 | 1 | *copA* | Multicopper oxidase type 3 | *Novosphingobium aromaticivorans* |
|  | YP_498198.1 | 1 | *copA* | Multicopper oxidase type 3 | *Novosphingobium aromaticivorans* |
|  | YP_001714980.1 | 1 | *copB* | Copper resistance protein B precursor | *Acinetobacter baumannii* |
|  | YP_002128736.1 | 1 | *copB* | Copper resistance protein B precursor | *Phenylobacterium zucineum* |
|  | NP_747480.1 | 1 | *copB* | Copper resistance protein B precursor | *Pseudomonas putida* |
|  | YP_003391634.1 | 1 | *copB* | Copper resistance protein B precursor | *Spirosoma linguale* |
|  | CBW99319.1 | 1 | *copB* | Copper resistance protein B precursor | *Legionella pneumophila* |
|  | ZP_08142794.1 | 1 | *copB* | Copper resistance protein B precursor | *Pseudomonas* sp. |
|  | YP_004535038.1 | 1 | *copB* | Copper resistance protein B precursor | *Novosphingobium* sp. |
|  | CCC56739.1 | 1 | *copB* | Copper resistance protein B precursor | *Weissella thailandensis* |
|  | YP_004825192.1 | 1 | *copB* | Copper resistance protein B precursor | *Rhodothermus marinus* |
|  | ZP_08952862.1 | 1 | *copB* | Copper resistance protein B precursor | *Rhodanobacter* sp. |
|  | YP_497408.1 | 1 | *copB* | Copper resistance protein B precursor | *Novosphingobium aromaticivorans* |
|  | YP_498199.1 | 1 | *copB* | Copper resistance protein B precursor | *Novosphingobium aromaticivorans* |
|  | YP_498200.1 | 1 | *copB* | Copper resistance protein B precursor | *Novosphingobium aromaticivorans* |
|  | ZP_01303665.1 | 1 | *copB* | Copper resistance protein B precursor | *Sphingomonas* sp. |
|  | ZP_01303683.1 | 1 | *copB* | Copper resistance protein B precursor | *Sphingomonas* sp. |
|  | YP_001899286.1 | 1 | *copC* | Copper resistance protein | *Ralstonia pickettii* |
|  | YP_002130323.1 | 1 | *copC* | Copper resistance protein | *Phenylobacterium zucineum* |
|  | ZP_03543176.1 | 1 | *copC* | Copper resistance protein | *Comamonas testosteroni* |
|  | ZP_04661420.1 | 1 | *copC* | Copper resistance protein | *Acinetobacter baumannii* |
|  | YP_002983463.1 | 1 | *copC* | Copper resistance protein | *Ralstonia pickettii* |
|  | ZP_05361746.1 | 1 | *copC* | Copper resistance protein | *Acinetobacter radioresistens* |
|  | ZP_06692649.1 | 1 | *copC* | Copper resistance protein | *Acinetobacter* sp. |
|  | ZP_08071275.1 | 1 | *copC* | Copper resistance protein | *Methylocystis* sp. |
|  | ZP_08389878.1 | 1 | *copC* | Copper resistance protein | *Sphingomonas* sp. |
|  | ZP_08390470.1 | 1 | *copC* | Copper resistance protein | *Sphingomonas* sp. |
|  | YP_497413.1 | 1 | *copC* | Copper resistance protein | *Novosphingobium aromaticivorans* |
|  | YP_949428.1 | 1 | *copD* | Copper resistance protein | *Arthrobacter aurescens* |
|  | NP_601654.1 | 1 | *copD* | Copper resistance protein | *Corynebacterium glutamicum* |
|  | ZP_03821649.1 | 1 | *copD* | Copper resistance protein | *Acinetobacter* sp. |
|  | YP_003020097.1 | 1 | *copD* | Copper resistance protein | *Geobacter* sp. |
|  | ZP_06064061.1 | 1 | *copD* | Putative copper export protein | *Acinetobacter johnsonii* |
|  | ZP_07047701.1 | 1 | *copD* | Copper resistance protein | *Comamonas testosteroni* |
|  | YP_003917755.1 | 1 | *copD* | Copper resistance protein | *Arthrobacter arilaitensis* |
|  | AAT07758.1 | 1 | *copF* | CopF Cu-ATPase | *Xanthomonas perforans* |
|  | YP_002890929.1 | 1 | *copK* | Copper resistance protein | *Thauera* sp. |
|  | YP_145674.1 | 1 | *copL* | Putative type II restriction enzyme | *Cupriavidus metallidurans* |
|  | YP_001338357.1 | 1 | *cusR* | Copper resistance transcriptional regulator | *Klebsiella pneumoniae* |
|  | YP_002961462.1 | 1 | *cusR* | Copper resistance transcriptional regulator | *Methylobacterium extorquens* |
|  | NP_754181.1 | 1 | *cutC* | Copper homeostasis protein | *Escherichia coli* |
|  | YP_001899292.1 | 1 | *pcoD* | Putative copper export protein | *Ralstonia pickettii* |
|  | YP_113307.1 | 1 | *pcoD* | Putative copper export protein | *Methylococcus capsulatus* |
|  | YP_001485623.1 | 1 | Others | Copper export protein | *Bacillus pumilus* |
|  | YP_002553639.1 | 1 | Others | Copper resistance outer-membrane lipoprotein | *Acidovorax ebreus* |
|  | ZP_05136509.1 | 1 | Others | Response regulator in two-component regulatory system with CusS, regulation of copper resistance | *Stenotrophomonas* sp. |
|  | YP_002947543.1 | 1 | Others | Putative copper resistance-related lipoprotein | *Variovorax paradoxus* |
